# Where does a ‘foreign’ accent matter? *German, Spanish and Singaporean listeners’ reactions to Dutch-accented English, and standard British and American English accents*

**DOI:** 10.1371/journal.pone.0231089

**Published:** 2020-04-29

**Authors:** Warda Nejjari, Marinel Gerritsen, Roeland van Hout, Brigitte Planken

**Affiliations:** Department of Language and Communication, Radboud University, Nijmegen, The Netherlands; Aix-Marseille Université, FRANCE

## Abstract

How well L2 English is understood and how L2 English speakers perceive one another within varying communication contexts has been studied relatively rarely, even though most speakers of English in the world are L2 speakers. In this matched-guise experiment (N = 1699) the effects of L1 and L2 English accents and communication context were tested on speech understandability *(intelligibility*, *comprehensibility*, *interpretability)* and speaker evaluations *(status*, *affect*, *dynamism)*. German (N = 617), Spanish (N = 540), and Singaporean listeners (N = 542) were asked to evaluate three accents (Dutch-accented English, standard British English, standard American English) in three communication contexts (Lecture, Audio Tour, Job Pitch). The main finding is that the Dutch-accented English accent was understood as well as the two L1 English accents. Furthermore, Dutch-accented English evoked equally positive evaluations to the two L1 English accents in German listeners, and more positive evaluations than the two L1 English accents in Spanish and Singaporean listeners. These results suggest that accent training aimed at achieving an L1 English accent may not always be necessary for (Dutch) English language learners, especially when they are expected to mostly interact with other L2 speakers of English. More generally, our results indicate that L2 English speakers’ understanding and their evaluation of L1 and L2 Englishes would not seem to reflect traditional language norms. Instead, they seem to reflect the socio-cultural embedding of a language norm in a Lingua Franca English speech community that does not view accent varieties as a hindrance to successful communication.

## Introduction

Traditionally, in second language acquisition (SLA) research the perspective is that fully acquiring an L2 involves achieving proficiency in a ‘target language’, free from L1 influence [e.g. 1–7], which most L2 speakers do not achieve [4–6, 8]. This perspective has been criticized for assuming that L2 speakers are at a linguistic disadvantage because they are less ‘competent’ than L1 speakers and for not sufficiently taking the social and interactive nature of language use into consideration [e.g. 9, 10]. With respect to the latter, for example, Firth and Wagner [9] have called for a more holistic view on SLA. They argue that it is important to acquire more knowledge on the social context, the interactive nature of language acquisition, and the effects of language use on the perceptions of speakers.

An alternative position on SLA is offered by Canagarajah [11] who argues that the current position of English as a global lingua franca illustrates that the traditional SLA perspective is not optimal. Most speakers of English in the world are L2 speakers with various cultural and linguistic backgrounds [12] who communicate with one another in a variety of societal domains, such as business, politics, and academia. Canagarajah suggests that speakers who use English as a global lingua franca effectively form a worldwide speech community of their own, an international group of L2 English speakers, not separated by traditional national and linguistic boundaries, who view English as a resourceful tool to achieve their objectives [13, 14]. At the same time, individual L2 English speakers in this community are members of their own linguistic communities, for example, within nation states. This means that so-called ‘Lingua Franca English (LFE) speech community members’ can have multiple linguistic identities, and that linguistic heterogeneity and flexibility are the norm within their community. Consequently, community members are probably less focused on how fellow L2 English speakers are dissimilar to L1 English speakers and how that may potentially hinder communications and reflect (potentially negatively) on how they are perceived as speakers.

Canagarajah’s assumption of the existence of an LFE speech community and its members’ potential flexible language attitudes is to some extent supported by speech evaluation research that has shown that L2 English language users can be as tolerant or more tolerant of an L2 English variety compared to L1 English varieties [15]. At the same time, L2 English language users have been found to evaluate L2 Englishes more negatively than L1 varieties [15–20]. Even though there has been some research on the effects of and attitudes towards L1 and L2 English varieties [e.g. 21–35], more knowledge is required on the responses to different varieties of English in order for learners, but also teachers, to navigate the new linguistic reality of an LFE speech community in which multiple varieties of English co-exist.

The current study takes Dutch-accented English as an example of L2 English to assess how global LFE speech community members with different L1 backgrounds (than Dutch) understand and perceive L2 English compared to varieties of L1-accented English (British and American).

### Dutch English in the LFE speech community

In this study, Dutch-accented English was selected as an example of L2 English that might illustrate the existence of the LFE speech community. As the most dominant L2 language in Dutch education, English is learned by all Dutch secondary school pupils and the majority of primary school pupils [35–38]. All secondary school pupils finish their secondary education with an English exam that tests reading, listening, writing and speaking [37, 38]. English is also the most important L2 language in advertising, academia, politics, and business and the most important lingua franca in transnational communications [e.g. 35, 36]. Some have even argued that English has integrated into Dutch society to such an extent that it is gaining a status similar to the English spoken in former British colonies, such as Singapore, Nigeria, and India [see 39].

However, despite the widespread use of English in the Netherlands, and the fact that, generally, Dutch speakers of English are considered proficient L2 speakers of English [e.g. 35; ranked nr. 2 out of 88 nations according to 44; 37, 38], there appear to be negative perceptions in Dutch society regarding the impact of Dutch accentedness in English on effective international communication, but also on English use within specific Dutch contexts. For example, in Dutch higher education degree programmes are increasingly offered in English. Some have criticized the English language skills of Dutch lecturers in these degree programmes, in that they argue that Dutch influenced English, for instance at the level of pronunciation, might hinder knowledge transfer [e.g. 40–43]. Such assumptions have to some extent been confirmed by Hendriks, van Meurs and Hogervorst [24]. They found significantly more negative speech understanding evaluations by Dutch students’ when a lecture was taught by a lecturer with a strong Dutch English accent compared with a lecturer with a slight Dutch English accent, or a lecturer teaching in Dutch.

Hendriks, van Meurs and Hogervorsts’ [24] findings, and the other anecdotal negative perceptions in the Netherlands of Dutch-accented English [40–44], might be taken to reflect a general language norm in Dutch society based on the view that proficiency as an L2 English speaker is only truly achieved when a speaker’s language skills match those of an L1 English speaker. This corresponds with the traditional SLA perspective. However, it might not reflect how Dutch-accented English is actually understood and perceived within the global LFE speech community where varieties of L2 Englishes are used internationally in interactions between speech community members with diverse cultural and linguistic identities [e.g. 11]. It is therefore relevant to determine how Dutch speakers’ English use affects their interactions with specific L2 speaker groups from other countries (than the Netherlands) who are active in the domains in which English is used in the larger LFE community. The L2 English speaker groups that were selected for this experiment are Germans, Spaniards and Singaporeans, as these speakers are from countries that are important to the Netherlands in terms of trade, politics, and academia.

Germany, which neighbors the Netherlands, is its most important trade partner [45, 46]. Like Dutch, Germany’s only official language, German, is a West-Germanic language, and English is spoken in Germany at a high level [ranked as one of the nations that is most fluent in English: at number 10 of 88 nations, 44]. This means that the linguistic distance between Dutch and German L2 English speakers might be smaller compared to, for instance, the distance between Dutch and Spanish L2 English speakers, another important trade partner to the Netherlands [45, 47–49]. Spain has Spanish, a Romance language, as its official language (next to other official, regional languages: Basque, Catalan, and Galician), which is relatively more distant (than German) from Dutch. In addition, Spain does not have a high English fluency level and faces challenges in the effectiveness of their foreign language education [ranked at number 34 of 88 nations tested, 44; 50]. Taken together, the greater linguistic distance between Dutch and Spanish, the likely lack of familiarity of Spaniards with Dutch, and the differences between the Netherlands and Spain in English fluency levels might negatively affect the ability of Spanish L2 English speakers to understand Dutch-accented English.

Singapore, an important trading partner to the Netherlands as well [51], presents an interesting contrast to the Netherlands, Germany, and Spain regarding the status of English. It is an island state populated primarily by three ethnic groups with their own linguistic backgrounds (Chinese:73%, Malays:13.3%, and Indians: 9.1%) that have been using English as an L2 for centuries and are considered highly fluent in it [ranked at number 3 of 88 nations, 44, 52]. The dominant position that English as an L2 has acquired in Singapore, originally due to British colonialization, has led to its formal recognition as a national language in Singapore, in addition to Malay, Mandarin Chinese, and Tamil. English in Singapore has gone through a process of ‘nativization’, which means that the presence and use of English in Singaporean society has been intense enough to develop into a variety commonly referred to as Singaporean English that is marked by a pronunciation, vocabulary, and grammar that is distinguishable from L1 and other L2 varieties of English.

Varieties like Singaporean English are mostly not defined as L1 Englishes similar to, for example, British or American English because they did not originate in the nations that, from a western and European perspective, were considered the cultural and linguistic centers from which English emerged and developed during the British colonial period. In addition, such varieties are not norm-providing in the sense that they are not used as a model for learners of English [10, 53, 54]. Indeed, while an increasing number of Singaporeans view English as their L1 [52, 54], and English is an official language in Singaporean education, academia, law, government, and business, it has a separate, functional, status from Malay, Mandarin or Tamil. The latter are languages that mark Singaporeans’ cultural identity (Malay, Chinese or Indian) and are mostly used in non-formal, private contexts [52, 55]. This means that for Singaporeans dealing with speakers of English with various cultural and linguistic identities is commonplace [56]. As a result, Singaporeans might have developed a more flexible language norm and do not necessarily view L2 English speech more negatively nor find it less understandable than L1 English speech. However, such assumptions have not been empirically investigated. Therefore, studying how Singaporean English speakers respond to L2 English accentedness (in our case Dutch-accented English) compared to L2 English speakers such as Germans and Spaniards can offer interesting insights into how different English speaker groups, and LFE community members, understand and evaluate Dutch-accented L2 English.

### Speech understandability

If an LFE speech community [11], with its own language norms and attitudes, does indeed exist, it needs to be established how well L2 English varieties produced by different speaker groups in that community are understood. A considerable number of studies have researched the degree to which speech is (perceived to be) understood [e.g. 24, 57–66]. Most have assessed understanding in terms of a single distinct level of understanding speech, for example, through orthographic transcription [e.g. 65–66] which reflects the degree to which listeners are able to determine individual words, or asking content questions to test whether content has been understood and to what extent [e.g. 64].

The different components of speech understanding are generally not assessed in one experiment and therefore it is not clear yet how L1 and L2 English accents impact different speech understanding components. An exception is by Nejjari et al. [27, 28] who operationalized Kachru and Smith’s [67] concept of speech understanding or *speech understandability* as a process consisting of three components. The first component, *intelligibility*, refers to how utterances are deciphered into individual sound patterns that form words and sentence-level elements. *Intelligibility*, as mentioned above, can be measured by asking listeners to orthographically transcribe individual words or sentences produced by speakers [see also 27, 65, 66,68]. The second component, *comprehensibility*, refers to the ability to understand the individual meaning of words and how words put together express meaning within a specific context [see 27, 28]. *Comprehensibility* can be measured by asking listeners content questions on speech samples. The third component is *interpretability*, which is difficult to distinguish from *comprehensibility*, because both deal with meaning beyond recognition of sound patterns that form words and phrases [67, 27, 28]. *Interpretability* refers to whether listeners are able to correctly deduce a speaker’s intentions and the purpose of a communicative act [see 27, 28]. *Interpretability* can be measured by asking listeners about the intent of the speaker or about the communicative ritual that is taking place. Nejjari et al. [27] have shown that Dutch-accented English hindered *intelligibility*, but not *comprehensibility* and *interpretability* for British listeners; however, when this experiment was replicated with Dutch listeners, the three components of *speech understandability* were not negatively affected by Dutch-accented English [28]. In order to study in more fine-grained detail to what extent Dutch-accented English is understood by Germans, Singaporeans, and Spaniards, the three components of *speech understandability* were also measured in the current experiment.

### Speaker evaluations

In addition to studying speech understanding it is also relevant to study the *speaker evaluations* L2 English accents arouse in terms of speaker characteristics, such as intelligence, friendliness or assertiveness. Some studies have shown that *speaker evaluations* of L2 accented English are not necessarily more negative compared to *speaker evaluations* of L1 accented speech in terms of *affect* (e.g. friendly, likeable) or *dynamism* (e.g. enthusiastic, proactive) [e.g. 24, 27, 28, 61, 62]. At the same time, other studies have shown that L2 (and L1) speakers of English tend to ascribe speakers of L2 English lower *status* (e.g. intelligent, cultured, competent) [e.g. 23, 29–34, 18, 67]. These varying findings might be explained by the L2 listeners’ linguistic circumstances, or the status and use of languages in individual societies. For example, Singaporeans’ specific linguistic circumstances (see earlier) might lead them to assign less negative *speaker evaluations* on the basis of Dutch-accented English than Germans and Spaniards. As was noted earlier, Singaporeans might be more used to and more accepting of various English accents and varying English fluency levels. Thus, providing more insight into the influence of language use on *speaker evaluations* beyond understandability, reflecting a more holistic view of SLA (see also above), would seem particularly relevant in an LFE community.

### Communication context

L2 English is not only spoken across and within L2 English speaker groups with diverse L1 backgrounds, it is also used to communicate in various communication contexts. These contexts have specific purposes and are linked to expectations in terms of (non-verbal and verbal) communicative behavior. For instance, the expectations for (non-verbal and verbal) communicative behavior of a lecturer in a lecture context may differ from the expectations for (non-verbal and verbal) communicative behavior of an interviewee in the context of a job interview. Furthermore, the topic and content discussed in a context may vary, and like context, can also impact (non-verbal and verbal) communicative behavior expectations. For example, a lecture context can have different topics, such as Dutch colonial history or disease patterns, and as a result vary in terms of content, which refers to what is stated about a topic and how. These topic and content variations can lead to specific communication expectations within a context; an audience might expect a history lecturer to discuss specific events and use objective language when discussing a controversial episode of colonial history. In short, communication context, topic and content are inextricably linked to one another and if expectations concerning them are violated, in a negative or positive sense, this may affect perceptions, and may even impact behavior during and after interactions [90]. The process of negative or positive expectancy violations has been suggested in Expectancy Violation Theory [69, 70], where it applies more specifically to non-verbal communication behavior in, for example, intercultural personal communication contexts. If we extend the idea of expectancy violations to accent production in an LFE speech community setting, it might explain why evaluations of accents and speakers can vary across communication contexts. It might be the case that certain accents (L1 or L2) are (un)expected or deemed (un)desirable in specific communication contexts. Cargile [32] investigated American (Anglo- and Asian American) listeners’ evaluations of Mandarin-accented English compared to standard American-accented English in a higher education lecture and a job interview context. These communication contexts can be considered high stakes contexts, even if topic and content vary, since not understanding a speaker’s accent in these contexts might mean that topic and content are not transferred effectively. For example, in the case of a lecture, students might not be able to perform optimally on tests as a result, while evaluating an interviewee negatively on the basis of their accent may result in that person not being hired for a position. Cargile [32] found that listeners made no distinction between standard American-accented English and Mandarin-accented English in a job interview context, but did so in a higher education lecture context, where the Mandarin-accented English aroused lower dynamism, status, and affect than the standard American English accent. Cargile, whose study involved students as listeners, suggests that listeners’ judgments might have been influenced by the fact that they were more familiar with the lecture context than the job interview context.

In another study that tested responses to accents in different communication contexts, Nejjari et al. [28] investigated Dutch listeners’ responses to Dutch-accented English compared to standard British and American English in three communication contexts that these listeners would likely be familiar with in international settings (education, business, tourism). The results showed that communication context impacts speaker evaluations. The speakers evaluated in a job pitch context were almost universally perceived more negatively, regardless of their accent, an effect which was not observed for the lecture and the audio tour. The results differ from those of Cargile [32], potentially because the listeners in Cargile [32] were L1 English speakers, and in Nejjari et al. [28] they were L2 English speakers.

The influence of context emerging from the few studies that have investigated its role in the evaluation of accentedness [28, 32] might be an indication that different language norms hold for different communication contexts, topic and content, as can be hypothesized based on Expectancy Violation Theory (see above). In the present study, therefore, communication context given its potential relevance, was included as a variable to compare responses by L2 English speakers to L2 and L1 English accents in different situations of use. Even though we realize that topic and content can also impact responses to speech, practical considerations have led us to only include context as a variable.

### Purpose of experiment

The current study investigated the reactions of listeners from Germany, Spain, and Singapore to L1 and L2 accented English. It featured Dutch-accented English and standard British and American English accents respectively. Both L1 Englishes are models generally used in English education for L2 English speakers around the world [71], and therefore function, from a traditional SLA perspective, as the ‘target’ language varieties that learners are encouraged to master in formal education. The idea is that if learners aim to all acquire the ‘target’ language, this will increase the likelihood that speakers with different L1 backgrounds will understand each other [e.g. 10, 72–74]. However, it remains unclear what the actual effects of L2 English accents are within the LFE speech community on different components of speech understandability and speaker evaluations, and in more than one communication context. This is the question the present study aimed to answer with respect to Dutch-English. If L2 English speakers in different countries do not necessarily share the traditional SLA perspective, and L2 English accents do not hinder speech understandability and speaker evaluations, this might mean that in terms of accentedness, achieving an L1 English accent is not necessary.

The present study investigated the response to Dutch-English (versus British and American) accent by L2 English groups from countries with varying reported average English fluency levels that are important partners to the Netherlands, namely Germany, Spain and Singapore. Furthermore, the groups have L1s that vary in relatedness to Dutch and/or English. While German, Dutch, and English are linguistically related to one another because they are West Germanic languages, Spanish is not as related to Dutch as German or English, since it is a Romance language. Singaporean listeners, in turn, speak a variety of English that is generally not considered an L1 English, although it is heavily influenced by British English [80]. This might mean that Singaporean English is linguistically more related to English, Dutch, and German compared to Spanish. In the present study, the different characteristics of the three listener groups were expected to lead to different responses to Dutch-accented English. For example, studies have shown that familiarity can aid *speech understandability* [27, 64, 75–79, 80]. As German listeners are likely to be more familiar with Dutch-accented English, because the Netherlands is a neighboring country to Germany, they are one of the closest political, economic and EU partners [81], and both have a West Germanic language as their official language and L1, they might, as a result, have higher *speech understandability* of Dutch-accented English compared to Spaniards or Singaporeans who are likely to be less familiar with Dutch and a Dutch English accent.

The mixed results from earlier studies with regard to the *speech understandability* and *speaker evaluations* of L2 English, and the limited research into the manner in which communication context might impact responses to L2 English [28, 32], led to the inclusion of three different communication contexts in our experiment: a lecture, a retail manager job pitch, and an art gallery audio tour. These contexts (and their associated topics and content) were selected to represent the settings in which L2 English is frequently employed: academia, international business, and tourism [28, 32, 36].

In order to determine whether *accent* and *context* affect *speech understandability* and *speaker evaluations*, two research questions and six expectations were formulated. The first research question was:

RQ1: Do German, Spanish, and Singaporean listeners (*listener group*) display different *speech understandability* (*intelligibility*, *comprehensibility*, *interpretability*) in response to Dutch-accented English compared to standard British and American English accents (*accent*), and does *context* (lecture; audio tour; job pitch) affect their responses?

Due to Spain’s reported lower English fluency level, and the fact that Spanish is typologically more distant from Dutch compared to German, it is expected that compared to German listeners, Spanish listeners will show lower *speech understandability* of Dutch-accented English, standard British and American English accents compared to German and Singaporean listeners. Therefore, the following expectation was formulated:

*Expectation 1a*: *Spanish listeners will display a lower level of speech understandability of the tested accents compared to German and Singaporean listeners*.

Singapore’s reported high English fluency, its official recognition of English as a national language, and its linguistically diverse population that regularly communicates across ethnic groups in English might facilitate *speech understandability* of the various English accents in the Singaporean listener group more than in the listener groups from the relatively homogenous societies in this study, namely Germany and Spain. Consequently, the following expectation was formulated:

*Expectation 1b*: *Singaporean listeners will display a higher level of speech understandability compared to German and Spanish listeners*.

Following Cargile [32] and Nejjari et al. [28], our experiment assessed responses to accents in a lecture, job pitch, and audio tour context. Only highly educated listeners were selected to assess the accents, since they represent the segment of the population that is most likely to be familiar with the three contexts. The communication context a highly educated listener was assumed to be most familiar with was the lecture context, because individuals who have followed high(er) education are very likely to have attended academic lectures. Relatively higher familiarity with the lecture context was expected to aid *speech understandability* in this context, because listeners were expected to recognize the lecture genre and the communicative rituals it entails, which could potentially allow them to more easily concentrate on the content of what is communicated. Therefore we expected that:

*Expectation 1c*: *The lecture communication context will evoke higher speech understandability compared to the job pitch and audio tour communication contexts*.

In addition to studying the effects of accent and context on *speech understandability*, their effects on *speaker evaluations* were assessed as well. It was assumed that the listener groups’ responses to the tested English accents might differ due to their diverse linguistic backgrounds and the status of English in their own societies. The listeners’ responses to L2 and L2 English accents, in turn, could be an indication of potentially different language norms. As a result, the second research question was:

RQ2: Do German, Spanish, and Singaporean listeners (*listener group*) display different *speaker evaluations* (*status*, *affect*, *dynamism*) in response to Dutch-accented English compared to standard British and American English accents (*accent*), and does *context* (lecture; audio tour; job pitch) affect their responses?

In general, speaker evaluation research has shown that L2 listeners ascribe L2 English accents lower status, but not lower affect and dynamism, compared to L1 English accents [e.g. 24, 27, 28]. This is why we expected that L2 English speakers from Germany and Spain would ascribe Dutch-accented English lower status than standard British and American English:

*Expectation 2a*: *German and Spanish listeners will ascribe Dutch-accented English lower status compared to standard British and American English accents*.

Singapore’s linguistically and culturally diverse population has developed its own nationally recognized variety of English, Singaporean English, which is generally not considered an L1 English. It was assumed that Singaporeans communicate with English speakers from various national and linguistic backgrounds, and that therefore, Singaporean listeners might not view accentedness as an important marker of a speaker’s character or abilities. As a result, they might not evaluate L2 and L1 English accents differently. This is why we expected that:

*Expectation 2b*: *Singaporean listeners will not display different speaker evaluations in response to Dutch-accented English compared to standard British and American English accents*.

Based on the results of a limited number of studies, it would seem that L1 and L2 listeners evaluate speakers with the same accent differently when the accent is presented in different communication contexts (*context)*. In the case of L1 English speakers as listeners (Anglo- and Asian Americans) [32], for example, a lecture aroused lower dynamism, status, and affect when produced with an L2 English accent compared to an L1 English accent. This effect was not observed in a job interview context. In a study by Nejjari et al. [28], involving the same contexts as the present study, and L2 English speakers as listeners (Dutch), a lecture context in L1 English accents did not result in lower *speaker evaluations* compared to a lecture context in an L2 English accent. However, the job pitch, regardless of accent, evoked significantly lower *speaker evaluations* compared to the lecture and audio tour. As the current study is also focused on L2 English listeners, albeit on three different groups of L2 English speakers (German, Spanish, Singaporean), we expect similar *speaker evaluation* patterns to emerge as in Nejjari et al. [28], resulting in the final expectation:

*Expectation 2c*: *The job pitch context will evoke lower speaker evaluations compared to the lecture and audio tour context*.

## Materials and methods

To investigate the effects of *listener group*, *accent* and *context* on *speech understandability* and *speaker evaluations*, a matched-guise experiment was conducted in which we compared three listener groups’ responses (N = 1699) from Germany (N = 617), Spain (N = 540), Singapore (N = 542) to three English accents (Dutch-accented English, standard British and American English) in three communication contexts (a lecture, an audio tour, a job pitch). All listeners responded to stimuli (speech samples) via an online questionnaire. The experiment had a within-subject multi-factorial design. All listeners (*listener groups*) were exposed to the independent variables (*accent*, *context*) and evaluated the stimuli on the dependent variables (*speech understandability*, *speaker evaluation*s).

### Speakers: Matched-guise speaker, control and filler speakers

To avoid participants responding to the voice characteristics of individual speakers, a matched-guise speaker was selected to produce the three accents (see S1 matched-guise speaker speech samples). The matched-guise speaker, who was a native speaker of Dutch, had been assessed in an earlier speech evaluation experiment [82], which showed that he could produce the three accents under study that represented the independent variable *accent*: (1) standard British English, (2) standard American English, and (3) the typical English accent of highly educated L1 speakers of Dutch. In the current study, the standard accents of British and American English refer to accents generally associated with the national accent norm of these nations and are generally similar to Received Pronunciation for a standard British English accent and General American for a standard American English accent. Dutch English does not have an explicit national norm. We regard a typical Dutch English accent in the present study as containing features that L1 speakers of Dutch and others familiar with Dutch and Dutch English will recognize as such. For example, because Dutch lacks dental consonants [ð] as in *this*, *mother*, *breathe* and [θ] as in *think*, *Martha*, *breath*, they are often mispronounced as stop consonants, [d] and [t] respectively, by Dutch speakers of English. Dutch also lacks voiced fricatives and plosives in the coda, causing the voiced coda obstruents of English to generally be pronounced as their voiceless counterparts in Dutch speakers’ English (e.g. *live*, *badge*, *bad*, *bag* will be said with [f, tʃ, t, k]) [83]. As no standard has been defined for a standard Dutch English accent, in this study it is defined as being ‘typical’ [28].

We included speech samples from six male control speakers as stimuli to prevent listeners from deducing that the study was focused on the matched-guise speaker: two L1 speakers of standard British English, two L1 speakers of standard American English, and two L1 speakers of Dutch who have a typical Dutch accent in English (see S1 control speech samples). All but one (a Dutch-accented control) had been assessed by L1 speakers of English as having a representative accent in our previous study [see 82]. However, the Dutch-accented English control speaker who was not assessed was regarded by experienced linguists as a representative speaker of Dutch-accented English. One further speaker produced a speech sample in a standard British English accent that was presented to listeners at the beginning of the experiment (the filler speech sample; see S1 filler speech sample) to familiarize them with the task. This filler speaker had also been assessed in our earlier study as an L1 and standard speaker of British English [82]. All speakers were aged 35 to 60 at the time of recording, had at least a master’s degree, and were English language and/or linguistics specialists in some capacity. The speech samples that were produced by the matched-guise speaker, the control speakers, and the filler speaker were compared for each context by the first author to ensure they did not deviate in terms of accent strength.

### Stimuli

One filler text (on a general topic) and three texts that represented the independent variable *context* were used as the basis for the speech samples: (1) an introduction to a marketing lecture; (2) an art gallery audio tour segment; (3) a job pitch for a retail management position (see S1 speech sample texts). All but the filler text reflect three contexts in which LFE is commonly used: higher education, tourism, and international business [35, 36, 39]. Due to practical reasons each listener had to evaluate all three communication contexts in the same order. The topic and content of the stimulus scripts in each context differed, to avoid exposing listeners to the same content three times and thus creating such familiarity with the content that this would influence their responses. This meant that the variable context includes topic and content, and thus potentially responses to the contexts were a result of the contexts’ varying topic and content. However, as indicated in the Introduction, context, topic and content are intertwined, because specific communicative expectations are indeed linked to communication context, but also to topic and content that could be expected or is commonly discussed within a specific context. In order to adequately measure responses to context, the lecture, audio tour, and filler texts were selected from an IELTS Academic English listening test and the job pitch text from a human resources webpage (see S1 speech sample links). This was to ensure that the content in the speech samples would be realistic, as natural as possible, and did not include politically sensitive or controversial information, to attempt to minimize the impact of the speech sample content on the listeners’ responses. The three communication contexts contained different topics and content. The topics per context were: (1) marketing in the lecture context, (2) aboriginal art in the audio tour, and (3) the position of retail manager in the job pitch. In addition, the content that was discussed was: (1) the definition of marketing in the lecture, (2) the work at display in the aboriginal art exhibition audio tour, and (3) the achievements of the interviewee for the position of retail manager in the job pitch. The matched-guise speaker produced the three accents in all three contexts, resulting in nine speech samples. The six control speakers produced their L1 accent in the three contexts, resulting in 18 samples. The filler speaker produced one speech sample on a general topic in standard British English. The speech samples were between 40 and 60 seconds long.

### Listeners: Age, education, L1 language(s), English fluency

Table 1 shows the listener groups’ sex, average age, their self-reported English fluency, and education level.

**Table 1:** Listeners (N = 1699): Age, % sex, self-reported English fluency, education level.

	Germany (N = 617)	Spain (N = 540)	Singapore (N = 542)
**Mean age:**	38 (Min = 19; Max = 83)	37 (Min = 18; Max = 64)	34 (Min = 18; Max = 80)
**Male: Female:**	46.0% 54.0%	34.3% 56.7%	44.9% 55.1%
**Mean self-reported English fluency (Min = 3; Max = 5)ᵃ**:	4.03	3.62	4.24
**Education level**ᵇ **Undergraduate: Bachelor: Master: Doctorate:**	0.0% 43.3% 52.0% 4.7%	0.0% 55.9% 39.4% 4.1%	0.0% 85.2% 12.8% 2.0%

ᵃMean self-reported English fluency was the mean for indicated levels for English listening, reading, writing, speaking skills on a 5-point scale. The mean had to be at least 3 in order to participate in the questionnaire (1: very low; 2: low; 3: average; 4: high; 5: like a L1 speaker).

ᵇThe original categories were: education at A-level, undergraduate; bachelor, master, PhD, other. Only listeners who had received an education at undergraduate level or above were allowed to participate in the questionnaire.

Highly educated listeners with at least average self-reported English fluency in terms of reading, listening, speaking, and writing were selected to represent the listeners who would most likely communicate in English in the selected communication contexts with other L2 speakers or L1 speakers of English. For example, in international business, which requires that people in the Netherlands, Germany, Spain and Singapore increasingly need to perform in English with international business contacts [36, 39–42].

### Instrumentation

Each listener evaluated four different speech samples by four different speakers (see S2): the filler sample followed by three experimental samples (in this order: Lecture, Audio Tour, Job Pitch) produced by the matched-guise speaker and the control L1 speakers. To ensure that the nine (plus 1 filler) matched-guise samples could be evaluated in each context for each accent, to avoid repeating the content of each context, and to limit any order effect, 18 listener groups were created. Data was collected from 18 listener groups, with a targeted 30 listeners per listener group in each country (see S2). This procedure and subsequent data quality checks (see also Data collection procedures) resulted in 617 completed questionnaires for Germany, 540 for Spain, and 542 for Singapore.

*Speaker evaluations*, *speech understandability* and the listeners’ estimation of the speakers’ country of origin were assessed for each speaker in the questionnaire followed by questions regarding the listeners’ English language skills, L1 languages, and general personal details. On the first page of the questionnaire the listeners were provided with a general introduction to the questionnaire but were not informed on the purpose of our study. All listeners first answered the *speaker evaluation* and *speech understandability* questions for the filler. They then answered the *speaker evaluation* and *speech understandability* questions for the Lecture, the Audio Tour, and the Job Pitch, always in this order. All listeners answered the *speaker evaluation* questions by first clicking on a link to the speech sample being evaluated (e.g. the Lecture), listening to the speech sample, and then answering the question (see S4-6). Next, on a separate page with no access to the speech sample link the listeners were asked to answer the *interpretability* and *comprehensibility* questions (see S4-6). Subsequently, on yet another separate page the listeners were asked to click on a link which led them to the recording of the first 10–12 words of the speech sample being evaluated, which they could listen to a maximum of two times in order to answer the *intelligibility* question (see S4-6). The questionnaire was designed in this manner to ensure that listeners would provide their first impression of the speakers’ traits (*speaker evaluations*) for each sample and only then display their understanding of the speech samples’.

As indicated, the listeners were also asked to indicate the speakers’ country of origin and did so after they had answered the *intelligibility* question. These answers were used to investigate whether correctly or incorrectly identifying the speaker’s accent affected *speaker evaluations* (see the Results and Discussion sections). For German listeners, 40.1% correctly indicated that the Dutch English speaker was from the Netherlands; for the Spanish and Singaporean listeners this was less than 2%. For each listener group, at least 87% recognized the standard British and American accent as L1 English accents. Dutch-accented English was recognized as an L2 English accent (i.e. not as specifically from the Netherlands) by a majority of the German (74%) and Singaporean (69%) listeners but by only a minority (42%) of the Spanish listeners.

#### Speech understandability

Following Smith and Nelson [84], Kachru and Smith [67], Nejjari et al. [27, 28], Bayyurt [85], and Berns [86] three questions were used to measure *speech understandability* and more specifically the ability (1) to literally recognize words (*intelligibility*), (2) to understand the meaning of the words within the context (*comprehensibility*), and (3) to understand the intention of the speaker / purpose of the message (*interpretability*) (see screenshots of questionnaire questions S4-6).

To measure *intelligibility*, listeners were presented with a speech sample consisting of the first 11–12 words of the three sample texts and asked to write down literally what was stated. If respondents were able to do so, this was counted as intelligible. Deviations in terms of spelling (e.g. ‘I’m’ instead of ‘I am’; ‘galery’ instead of ‘gallery’) or synonyms as transcriptions for the heard word were counted as correct (e.g. ‘good in developing’ instead of ‘strong in developing’). German and Spanish listeners at times also translated the English into their native language. When this occurred and the translation reflected the literal meaning of what was stated in the sample excerpt the translation was accepted, because the listeners would need to have been able to hear and understand the English correctly before being able to translate the English into German or Spanish. The listeners’ answers were analyzed in this way by the first author and three raters (native speakers of German, English, and Spanish) subsequently analyzed a subset of 20% of the listeners’ answers. An Intraclass Correlation Coefficient was calculated to measure interrater reliability and to establish the external consistency of the first author analyses compared to the raters’ assessments of the intelligibility analyses. It showed a correlation for each listener group of .91 (Germany), .97 (Spain), and .95 (Singapore) (0.00–1.00), which is extremely high. To measure c*omprehensibility*, listeners were asked to indicate whether a statement on the content of each sample was correct or not. *Interpretability* was measured by having listeners indicate whether a statement on the communicative intentions of the speaker of each sample was correct or not (see S4-6).

#### Speaker evaluations

To assess *speaker evaluation*s, listeners indicated on 5-point Likert scales (1 = strongly disagree; 5 = strongly agree; 3 = neither disagree nor agree) to which extent they believed the speaker possessed 11 personality traits. These traits represent three assumed dimensions of *speaker evaluation*, namely *status* (competent, educated, having authority, intelligent and cultured), *affect (*considerate, pleasant and friendly), and *dynamism* (energetic, enthusiastic, confident) (see S4-6). The traits associated with *status* and *affect* are based on Nejjari et al. [27]. *Status* represented the degree to which a speaker was viewed as being intelligent and well-educated, and *affect* represented the degree to which a speaker was perceived as being likeable. *Dynamism* measured the impression the speaker gave of himself, also known as a speaker’s ‘self-presentation’, and was based on Grondelaers and van Hout [87].

### Data collection procedures

The experiment was conducted in 2017 and 2018 via Qualtrics, a global survey software and online data collection company that caters for (non-)commercial organizations, and was originally launched for academics and the complex requirements of research [88]. In our case, Qualtrics was requested to sample listeners who were L1 speakers of the main national language of Germany (German), Spain (Spanish), and Singapore (Singapore English) and were highly educated (i.e. had at least reached or completed undergraduate level education). No restrictions were placed in terms of regions in the three countries where the experiment was circulated. At least 540 listeners per country were needed, and participants were registered individually. The agreement with the service also allowed for the main researcher to check the data and request replacement listeners in the case of inadequate responses.

Of the German listener data, 5% was initially collected in the context of a cross cultural communication research course at Radboud University in the Netherlands. Qualtrics was hired to collect the remaining 95% of the data. Subsequently, the listener data from Spain and Singapore was also collected by Qualtrics. On average, listeners needed 15 minutes to complete the questionnaire. For each country the data were checked by the first author, resulting in approximately 35% of the data being excluded. The reasons for excluding data included: listeners providing nonsense answers or numbers, symbols, or consistently providing neutral (mid-scale) answers only to all scale questions. All excluded data were replaced by new data which were checked again and replaced if necessary, resulting in a total of three rounds of data collection. Response analyses were conducted on the data collected for the matched-guise speech samples only.

### Statistics

Descriptives and frequencies were calculated to establish means and percentages of listener characteristics and responses (see S7 for the SURFdrive link to the datafile). We used ANOVAs when the dependent variable was continuous and logistic regression when it was binomial (two values only). We scrutinized residual scores to see whether we had serious violations of underlying statistical assumptions. There were no compelling reasons, given the robustness of both techniques, to apply alternative statistical techniques. The factor analyses we applied to trace underlying dimensions in speaker evaluations were principal component analyses, with varimax rotation.

## Results

### Speech understandability

This section will focus on whether German, Spanish, and Singaporean listeners display different *speech understandability* in response to Dutch-accented English compared to standard British and American English accents, and if context affects their responses (RQ1). With regard to our expectations, we will report whether the Spanish listeners displayed a lower level of *speech understandability* of the tested accents compared to German and Singaporean listeners (expectation 1a); whether Singaporean listeners displayed a higher level of *speech understandability* compared to German and Spanish listeners (expectation 1b); and whether the lecture communication context evoked higher *speech understandability* compared to the job pitch and audio tour communication contexts (expectation 1c). The frequencies and means for *speech understandability* (three accents, in three contexts) are presented in S8-10. A summary of the significant main and interaction effects are provided in Table 2.

**Table 2:** Summary results speech understandability (Intelligibility, Comprehensibility, Interpretability).

N = 1699	Intelligibility	Comprehensibility	Interpretability
**Listener Group (L)**	Germany > Singapore > Spain	NS	NS
**Accent (A)**	NS	NS	NS
**Context (C)**	NS	NS	NS
**L x A**	Germany: AE > BE Spain: AE > DE	NS	NS
**L x C**	Spain: LE > AT, JP	Germany: LE > AT, JP Singapore: LE, AT > JP Spain: AT > LE, JP Spain AT > Germany AT Germany JP > Singapore JP	Singapore: LE > JP Spain: AT, LE > JP Spain: AT > LE > JP Spain AT > Germany, Singapore AT Germany JP > Spain JP
**A x C**	NS	NS	NS
**L x A x C**	NS	NS	NS

ᵃA = Accent; AE = American English; AT = Audio Tour; BE = British English; C = Context; DE = Dutch English; JP = Job Pitch; L = Listener group; LE = Lecture; NS = Not Significant.

#### Intelligibility

**Fig 1:**
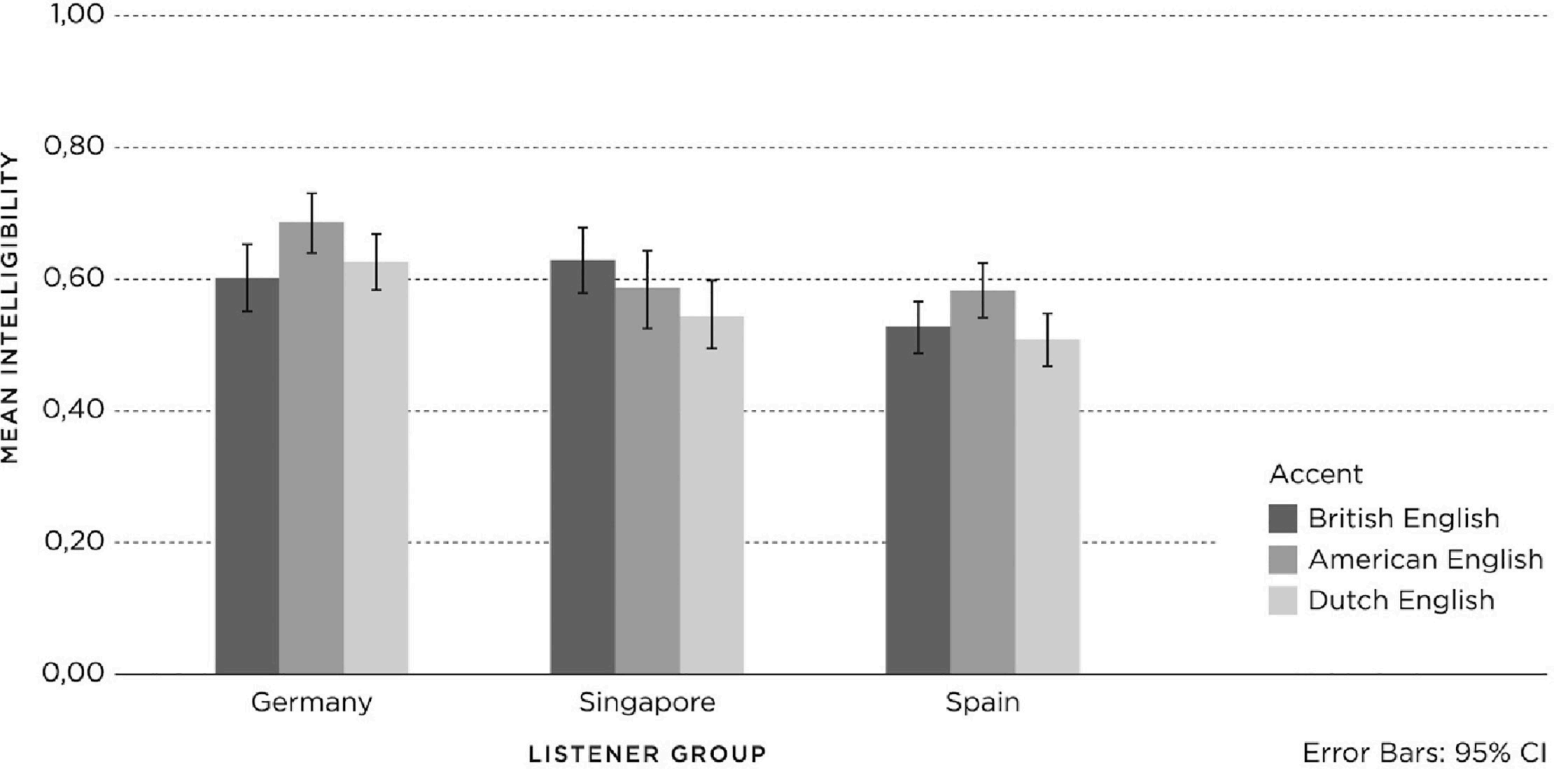
Mean proportions intelligibility for accent (British English, American English, Dutch English) and listener group (Germany, Singapore, Spain).

**Fig 2:**
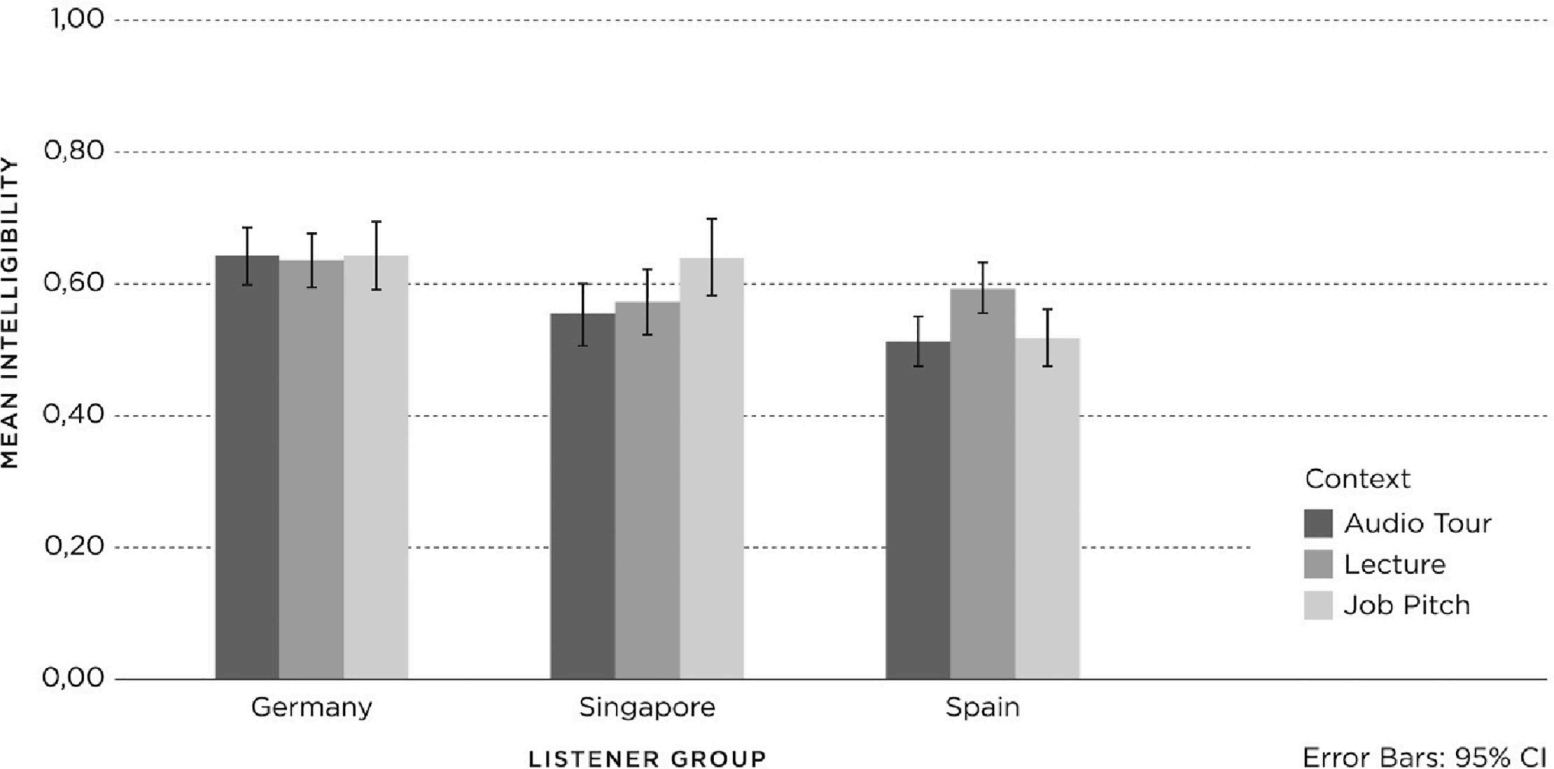
Mean proportions intelligibility for context (Audio Tour, Lecture, Job Pitch) and listener group (Germany, Singapore, Spain).

The listeners’ transcriptions of the first 11 to 12 words of each speech sample could result in a maximum of 11 (Lecture, Job Pitch) or 12 (Audio Tour) correctly transcribed words. Scores were equalized by computing the proportions of correct words. On average, the German listeners (N = 617) were able to correctly transcribe 7.47 (SD = 3.82) words, the Singaporean listeners (N = 542) 6.84 (SD = 4.08) words, and the Spanish listeners (N = 540) 6.14 (SD = 3.17) words. The results for *intelligibility* in relation to *accent* and *context* are shown in Figs 1 and 2, with 95% confidence intervals.

In terms of *accent*, there was substantial overlap between the confidence intervals of the bars, indicating that the responses to the three accents did not differ greatly between the three *listener groups* (Fig 1). In terms of *context*, there were substantial differences in the confidence intervals between and within the three *listener groups*, indicating that the responses to the three contexts differed more strongly (Fig 2).

A univariate analysis of variance was applied to investigate the effects of *listener group*, *accent*, and *context* on *intelligibility* and their interaction effects. The three-way interaction was not significant (F(8, 1672) = 1.449, p = .171, PES = .007), and neither was the two-way interaction between *accent* and *context* (F(4, 1672) = 0.167, p = .995, PES = .000). The two other interaction effects were significant though not strongly: *accent* by *listener group* (F(4, 1672) = 2.539, p = .038, PES = .006) and *context* by *listener group* (F(4, 1672) = 2.450, p = .044, PES = .006). The interaction effects can be interpreted by analysing the main effects, one of which, *context*, was not significant (F(2, 1672) = 2.344, p = .096, PES = .003). *Accent* (F(2,1672) = 4.704, p = .009, PES = .006) and *listener group* were significant (F(2, 1672) = 15.932, p = .000, PES = .019).

More details about the main effects were obtained through separate analyses of variance for each *listener group*, the factor involved in both significant two-way interactions. In the German group, effects were not significant for *context* (F(2, 608) = .148, p = .862, PES = .000), and *accent* by *context* (F(4, 608) = 1.315, p = .263, PES = .009). However, the effect of *accent* was significant (F(2, 608) = 3.679, p = .026, PES = .012), with a post-hoc difference (Tukey’s HSD) between *intelligibility* of American English (the highest score, .686) and British English (the lowest score, .602). In the Singaporean group, no significant effects were found (*accent*, F(2, 533) = 2.555, p = .079, PES = .009; *context*, F(2, 533) = 2.793, p = .062, PES = .010; *accent* by *context*, F(4, 533) = .342, p = .849, PES = .003). In the Spanish group, the two main effects and one interaction were significant (*accent*, F(2, 531) = 3.860, p = .022, PES = .014; *context*, F(2, 531) = 5.119, p = .006, PES = .019; *accent* by *context*, F(4, 531) = 1.901, p = .109, PES = .014). The post-hoc results for *accent* showed significantly higher *intelligibility* for American English (.584) than for Dutch English (.509). The post-hoc results (Tukey’s HSD) for *context* showed a significant difference in *intelligibility* between Lecture (.592) and the lower scores of Audio Tour (.511) and Job Pitch (.517).

The results show that there is no consistent hierarchy of accents and contexts for *intelligibility*; instead, they vary within the *listener groups*. No general effect was found for *accent*; however, when higher *intelligibility* was observed, it was for American English. Furthermore, Dutch-accented English never yielded the lowest *intelligibility*. Given the modest effects of *accent* and *context* on *intelligibility*, we conducted a post-hoc analysis (Tukey’s HSD) on the main effect of *listener group*, which yielded significant differences between the groups: Germans (.634) > Singaporeans (.588) > Spanish (.540), which showed the German listeners achieved highest scores for intelligibility, followed by the Singaporean and Spanish listeners.

#### Comprehensibility

**Fig 3:**
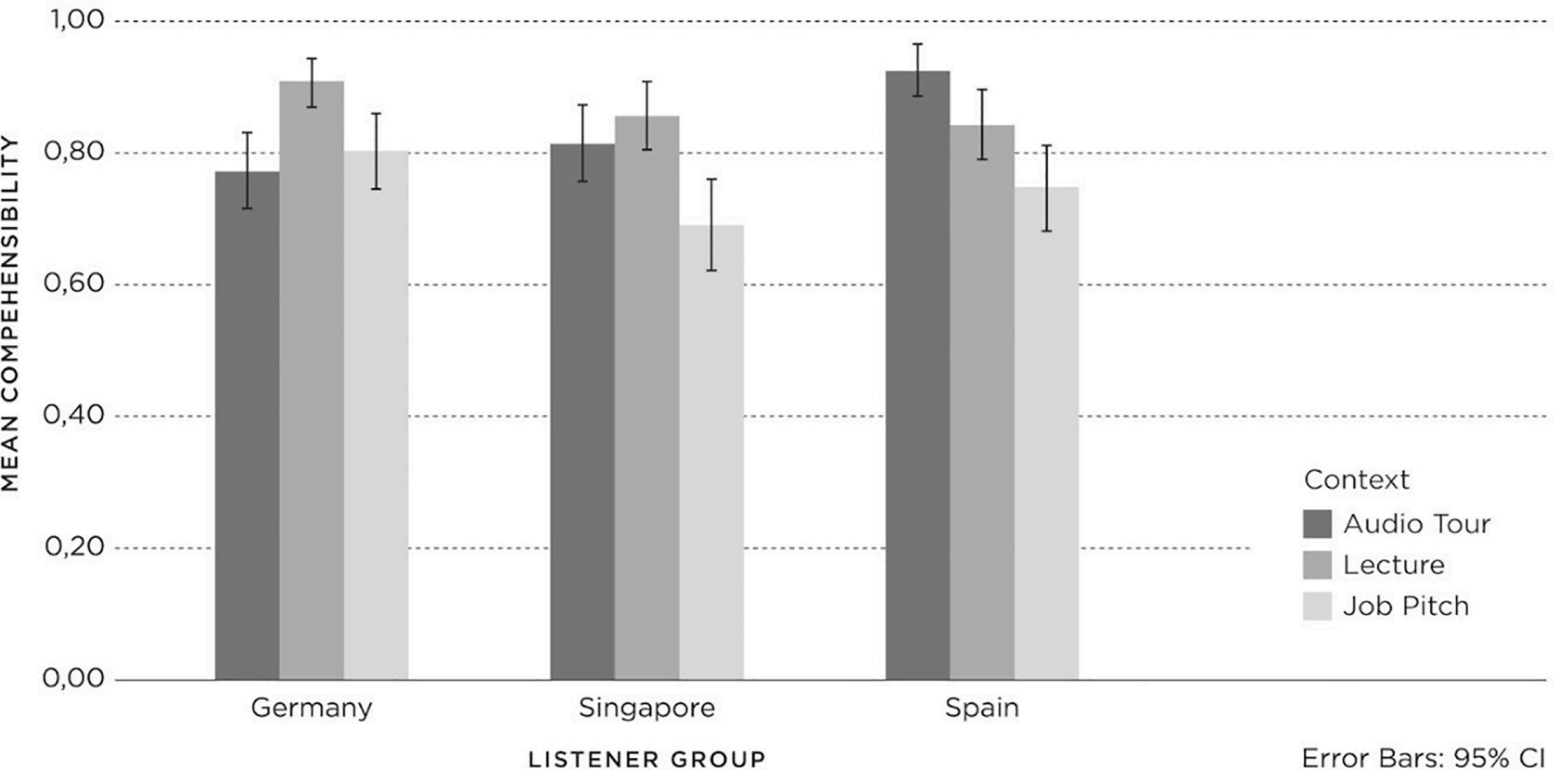
Mean proportions of correct comprehensibility for context (Audio Tour, Lecture, Job Pitch) and listener group (Germany, Singapore, Spain).

Of the Spanish listeners (N = 540), 84% correctly comprehended the speech samples, which for German listeners (N = 617) was 83% and for Singaporean listeners (N = 542), 79%. In terms of *accent*, the results demonstrated substantial overlap in the confidence intervals, indicating that there are no strong differences between the responses to the three accents per *listener group*, which is why no figure has been incorporated for *accent*. The results for *comprehensibility* in relation to *context* are shown in Fig 3, with 95% confidence intervals. In terms of *context*, there were more substantial differences in the confidence intervals between bars, indicating that the *comprehensibility* of the three contexts differed more strongly within each individual *listener group*.

A logistic regression was applied to test the effects of *listener group* (Germany, Spain, Singapore), *accent* (standard British English, standard American English, Dutch English), and *context* (Audio Tour, Lecture, Job Pitch), as well as their interaction. The final model contained significant effects of *listener group* (Wald = 6.063, df = 2, p = .048), *context* (Wald = 19.476, df = 2, p = .000) and their interaction (Wald = 22.997, df = 4, p = .000), but no significant effect of *accent*.

**Fig 4:**
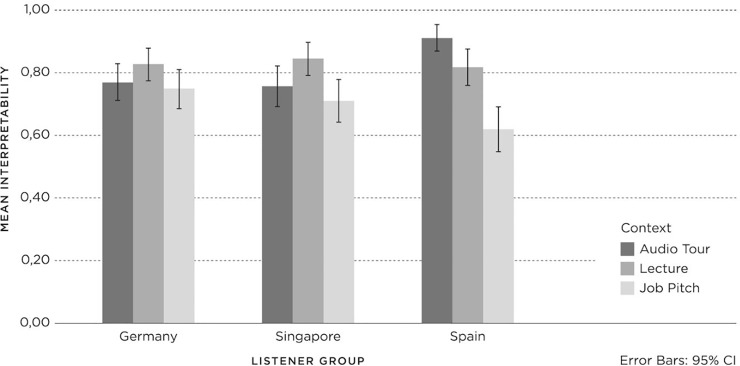
Mean proportions of correct interpretability for context (Audio Tour, Lecture, Job Pitch) and listener group (Germany, Singapore, Spain).

To investigate these effects in detail, we conducted a logistic regression per *listener group* with two pairwise comparisons (Bonferroni correction) to test the effect of context within each group. The German data returned a significant *context* effect (Wald = 14.643, df = 2, p = .001), with Lecture leading to significantly higher *comprehensibility* than Audio Tour and Job Pitch. The Singaporean data also returned a significant *context* effect (Wald = 15.701, df = 2, p = .000), but here it was the Job Pitch context that led to lower *comprehensibility* than the Audio Tour and Lecture contexts (see Fig 4). A significant context effect was also found for the Spanish group (Wald = 19.746, df = 2, p = .000), with *comprehensibility* being significantly higher in the Audio Tour context than in the Job Pitch context. In sum, the three contexts show varying results with respect to *comprehensibility* within the three *listener groups*. However, these are not strong, which means that, in general, there is no one context that is much more difficult or easier to comprehend than another within the three *listener groups*.

Next, we applied a logistic regression with two pairwise comparisons (Bonferroni correction) separately to the three contexts with *listener group* as predictor. For Audio Tour, there was a significant *listener group* effect (Wald = 15.884, df = 2, p = .000), with higher scores for the Spanish compared to Germans (see Fig 2). For Lecture, there was no significant difference between *listener groups* (Wald = 4.314, df = 2, p = .116). Job Pitch yielded a significant *listener group* effect (Wald = 6.063, df = 2, p = .048), but here *comprehensibility* was lower for the Singaporeans than for the Germans. In sum, this means that there is no general *listener group* effect on *comprehensibility*, and that differences between the *listener groups* do not depend on *accent*, but on *context*.

#### Interpretability

The German (N = 617) and Spanish listeners (N = 540) answered the *interpretability* question correctly in 78.3% of all cases, and the Singaporeans did so in 77.1% of cases (N = 542). In terms of *accent*, the results demonstrated substantial overlap in the confidence intervals, indicating that there are no strong differences between the responses to the three accents within each individual *listener group*, which is why no figure has been incorporated for *accent*. The results for *interpretability* in relation to *context* are shown in Fig 4, with 95% confidence intervals. In terms of *context*, there were more substantial differences in the confidence intervals between bars, indicating that the *interpretability* of the three contexts differed more strongly within each individual *listener group*.

A logistic regression was applied to test the effects of *listener group* (Germany, Spain, Singapore), *accent* (standard British English, standard American English, Dutch English), and *context* (Audio Tour, Lecture, Job Pitch), as well as their interaction. The final model selected only contained the effects of *listener group* (Wald = 7.292, df = 2, p = .026), *context* (Wald = 41.873, df = 2, p = .000) and their interaction (Wald = 23.715, df = 4, p = .000). There was no significant effect of *accent*.

To investigate these results in detail, we carried out a logistic regression with two pairwise comparisons (Bonferroni correction) per *listener group* to test the effect of context within each group. No significant *context* effect was found for the German data (Wald = 4.045, df = 2, p = .132). However, a significant *context* effect was found for the Singaporean data (Wald = 9.169, df = 2, p = .010), which turned out to be a single pairwise difference between Lecture and Job Pitch, with a higher score for Lecture (see Fig 4). The Spanish data also yielded a significant *context* effect (Wald = 41.873, df = 4, p = .000), with lower interpretability for the Job Pitch compared with the other two contexts (Lecture, Audio Tour). All pairwise comparisons for the Spanish listeners turned out to be significant, with a decreasing interpretability effect going from Audio Tour, Lecture, to Job Pitch (see Fig 4). In other words, the three contexts show varying results within the three *listener groups*, with none of the speech samples being generally more difficult or easier to interpret than the other ones across listener groups.

To evaluate the differences between the *listener groups* with regard to *interpretability*, we applied a logistic regression with two pairwise comparisons (Bonferroni correction) for each of the three contexts with *listener group* as predictor. For Audio Tour, there was a significant *listener group* effect (Wald = 16.175, df = 2, p = .000), with a significant difference between higher scores for Spain and lower scores for Singapore and Germany. For Lecture, no significant difference was found between *listener groups* (Wald = .433, df = 2, p = .805). Job Pitch yielded a significant *listener group* effect (Wald = 7.293, df = 2, p = .026), but here Spain was found to have the lowest score, which was significantly different from Germany, but not significantly different from Singapore. Overall, this means that there was no general effect of *listener group* nor of *accent* (Germany, Singapore, Spain) on *interpretability*, but that the differences between the *listener groups* depended on the *context* (Audio Tour, Lecture, Job Pitch) investigated.

#### Summary table speech understandability

Table 2 provides an overview of the results for *speech understandability* for the three tested accents and contexts for all three listener groups.

### Speaker evaluations

This section will report on the question whether German, Spanish, and Singaporean listeners display different *speaker evaluations* in response to Dutch-accented English compared to standard British and American English accents, and whether context affects their responses (RQ2). Furthermore, with regard to our three expectations, we will report whether German and Spanish listeners ascribed Dutch-accented English lower status compared to standard British and American English accents (2a); whether Singaporean listeners did not display different speaker evaluations in response to Dutch-accented English compared to standard British and American English accents (2b), and whether the job pitch context evoked lower speaker evaluations compared to the lecture and audio tour context (2c). A summary of the significant interaction effects, main effects, and post-hoc tests are provided in Table 4 at the end of this section. The mean and frequency measurements of the *speaker evaluations* of the three accents produced in three contexts are presented in S8-10.

A factor analysis, using a principle axis factoring extraction method with an Eigenvalue >1 criterion for factor extraction, followed by a varimax rotation, on the ratings of the personality traits that were measured to represent the three speaker evaluation constructs, showed a resolution into three factors for the German and Spanish *listener groups*, and two factors for the Singaporean *listener group* (see Table 3).

**Table 3:** Rotated Factor Matrix: Factor loadings scores on 11 scales with three factors for listener groups (Germany, Spain, Singapore). Only loadings >.550 are reported.

	Germany			Spain			Singapore	
	Factor 1	Factor 2	Factor 3	Factor 1	Factor 2	Factor 3	Factor 1	Factor 2
**Competent**	.736			.770			.732	
**Considerate**					.667			.609
**Cultured**	.759			.826			.713	
**Educated**	.803			.741			.828	
**Pleasant**		.693			.774			.668
**Energetic**			.830			.846		.812
**Authoritative**			.780			.749		
**Friendly**		.844			.829			.813
**Enthusiastic**		.641				.675		.815
**Intelligent**	.779			.756			.763	
**Confident**	.683			.670			.717	

*Status* was analyzed for the personality traits *competent*, *cultured*, *educated*, *intelligent*, and *confident*. The personality trait *authoritative* was excluded, because it was not perceived as similar enough to the other *status* personality traits by any of the three *listener groups* and did not yield loadings above .550 in the Singaporean *listener group*. *Affect* was analyzed for the personality traits *pleasant* and *friendly*, because all three *listener groups* evaluated these personality traits are part of one factor. The personality trait *considerate* was excluded because for German listeners it did not yield loadings above .550. *Dynamism* was analyzed for the personality trait *energetic* only, because for *listener groups* Germany and Spain this trait loaded as part of a third separate factor (see Table 3). This was not the case for Singaporean listeners, for which it was part of *affect*. Consequently, the Singaporean scores for affect resemble those of *dynamism*. It was nonetheless decided for all three listener groups to analyze it as part of *dynamism*. This was done in order to compare the responses to the trait *energetic* between the listener groups and see if there were different responses to the accents and contexts in terms of this personality trait. The personality trait *confident* was regarded as part of *status* by all *listener groups* and thus analyzed as such. The personality trait *enthusiastic* was excluded because German and Singaporean listeners regarded it as part of *affect*, and Spanish listeners as part of *dynamism*. In sum, on the basis of the loadings (see Table 3), three factors were defined and used in further analyses: *status (*competent, cultured, educated, intelligent, confident*)*, *affect* (pleasant, friendly*)*, *dynamism* (energetic).

#### Status

**Fig 5:**
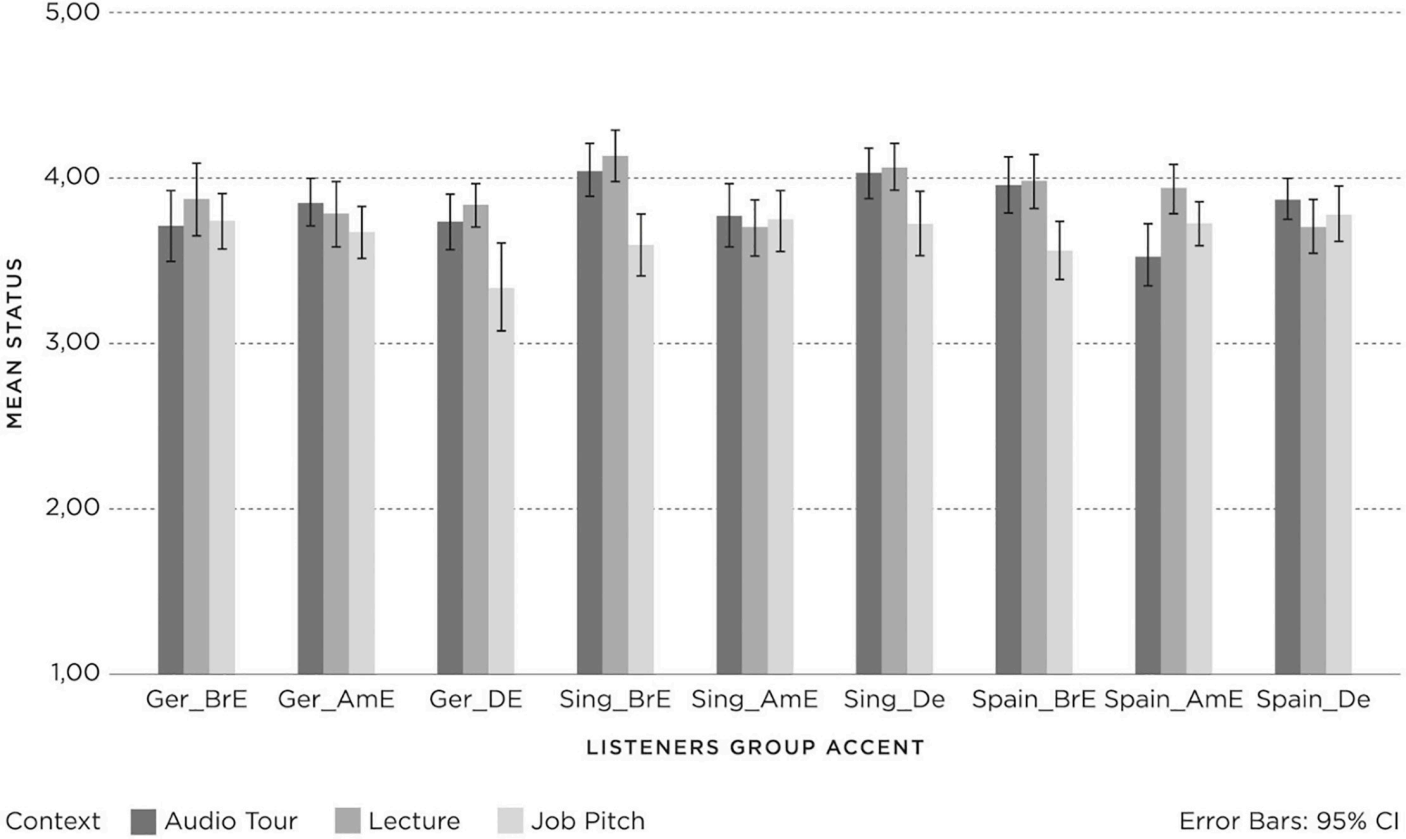
Status (1 = negative; 3 = neutral; 5 = positive) for Listener groups (Germany (Ger), Spain, Singapore (Sing)) per accent (British English (BrE), American English (AmE), Dutch English (DE)) and context (Audio Tour, Lecture, Job Pitch).

The results for *status* in relation to *listener group*, *accent* and *context* are shown in Fig 5, with 95% confidence intervals.

Univariate analyses of variance were applied and showed a significant three-way interaction for *listener group*, *accent* and *context* (F(8, 1672) = 4.30, p = .00, PES = .02) and significant two-way interactions for *accent* and *listener group* (F(4, 1672) = 2.98, p = .02, PES = .01), and *accent* and *context* (F(4, 1672) = 3.19, p = . 01, PES = .01). However, the interaction for *context* by *listener group* (F(4, 1672) = .69, p = .60, PES = .00) was not significant. Interpretations of the significant interactions must be seen in relation to the main effects. A significant main effect on status was found for *listener group* (F(2, 1672) = 6.26, p = .00, PES = .01) and *context* (F(2, 1672) = 18.61, p = .00, PES = .02), but not for *accent* (F(2, 1672) = 2.98, p = .051, PES = .00). Firstly, Post-hoc Tukey tests on *listener group* gave only one significant difference. The German listeners attributed significantly lower *status* to the speech samples than the Spanish listeners, and the Singaporean listeners did not differ in their *status* evaluations from the other two *listener groups*. For *context*, Job Pitch was evaluated significantly lower than Lecture and Audio Tour.

Subsequent univariate analyses were conducted within each *listener group* to investigate the effects of *accent* and *context* on *status* per *listener group*. For the German listeners, there were no significant two-way interaction effects for *accent* and *context* (F(4, 616) = 2.18, p = .07, PES = .01), and no main effect for *accent* (F(2, 616) = 4.16, p = .10, PES = .01). However, there was a main effect for *context* (F(2, 541) = 4.16, p = .02, PES = .02). Post-hoc comparisons (HSD) showed significantly lower *status* for the speaker giving the Job Pitch, compared to Lecture and Audio Tour. Interestingly, the German listeners who had indicated when they listened to Dutch-accented English that this was a non-native accent (m = 3.54; SD = .79) ascribed significantly lower status to the speaker compared to listeners who indicated that the Dutch-accented English was native (m = 4.04; SD = .53) (F(1, 216) = 4.34, p = .04, PES = .02).

For Singaporean listeners, there was a significant two-way interaction effect for *accent* and *context* (F(4, 541) = 6.46, p = .00, PES = .05), and a main effect for *context* (F(2, 541) = 4.16, p = .02, PES = .02), but there was no main effect for *accent* (F(2, 541) = 1.23, p = .29, PES = .01). Post-hoc comparisons (HSD) showed significantly lower *status* for Job Pitch (m = 3.68; SD = .62) compared to the Lecture (m = 3.88; SD = .61) but not the Audio Tour (m = 3.79; SD = .65). The two-way interaction for *accent* and *context* effect can be attributed to the different judgements on the Audio Tour for the American English accent; Audio Tour received lower scores than the other two contexts.

For the Spanish listeners, there was a significant two-way interaction effect for *accent* and *context* (F(4, 540) = 3.34, p = .01, PES = .03), and a main effect for *context* (F(2, 540) = 10.21, p = .00, PES = .04) and *accent* (F(2, 540) = 5.40, p = .01, PES = .02). Post-hoc comparisons (HSD) showed significantly lower *status* for American English compared to Dutch English and British English. For *context*, post-hoc comparisons (HSD) showed significantly lower *status* for the speaker giving the Job Pitch compared to the Lecture and the Audio Tour. The two-way interaction effect for *accent* and *context* can be attributed to the absence of context differences in relation to the American English accent (Tukey’s HSD).

However, in the Spanish *listener group* who listened to British English, the Job Pitch (m = 3.59; SD = .72) yielded the lowest speaker *status* compared to the Audio Tour (m = 4.05; SD = .60) and Lecture (m = 4.13; SD = .72), which was similar to the results for the Spanish *listener group* listening to Dutch English (Job Pitch m = 3.72, SD = .74; Audio Tour m = 4.03, SD = .59; Lecture m = 4.06, SD = .54). Finally, the Spanish listeners’ evaluations of the nativeness of the standard British English accent and *context* showed a significant two-way interaction effect (F(2, 174) = 3.53, p = .03, PES = .04). Spanish listeners who believed the standard British English accent was non-native (m = 3.91; SD = .62) in the Job Pitch context ascribed significantly higher *status* to the speaker compared to listeners who indicated that the British English accent was native (m = 3.46; SD = .72).

#### Affect

**Fig 6:**
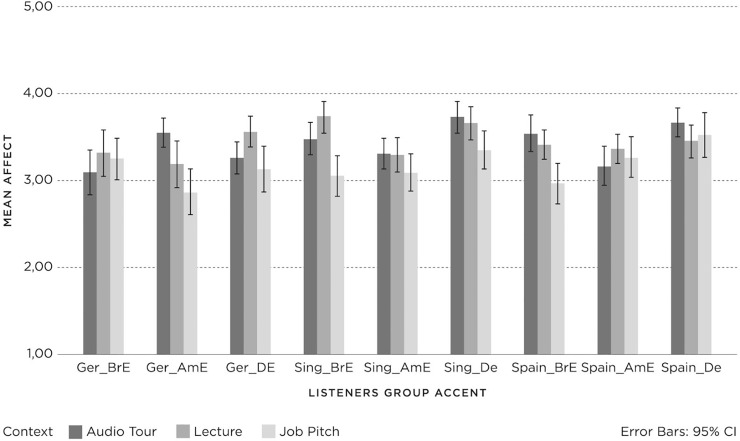
Affect (1 = negative; 3 = neutral; 5 = positive) for Listener groups (Germany (Ger), Spain, Singapore (Sing)) per accent (British English (BrE), American English (AmE), Dutch English (DE)) and context (Audio Tour, Lecture, Job Pitch).

The results for *affect* per *listener group*, *accent* and *context* are shown in Fig 6, with 95% confidence intervals. Univariate analyses of variance were applied to investigate the effects of *listener group*, *context* and *accent* on *affect* and their interaction.

The three-way interaction between *listener group*, *accent* and *context* (F(8, 1672) = 5.11, p = .00, PES = .02) was significant. None of the two-way interactions were significant: *accent* and *context* (F(4, 1672) = .87, p = . 48, PES = .00), *accent* and *listener group* (F(4, 1672) = 1.30, p = .27, PES = .00), and *context* and *listener group* (F(4, 1672) = 1.02, p = .40, PES = .00). The three main effects were all significant: *listener group* (F(2, 1672) = 5.99, p = .00, PES = .01), *accent* (F(2, 1672) = 12.83, p = .00, PES = .02), and *context* (F(2, 1672) = 18.69, p = .00, PES = .02). Firstly, Post-hoc Tukey tests showed that Dutch English evoked significantly higher *affect* than both British and American English. Secondly, German listeners attributed significantly lower *affect* to the speaker than the Singaporean and the Spanish listeners. Thirdly, the *affect* towards the speakers in the Job Pitch context was significantly lower compared with the *affect* towards the speakers in the Lecture and the Audio Tour contexts.

Subsequent univariate analyses were conducted within each *listener group* to investigate the effects of *accent* and *context* on *affect* per *listener group*. For the German listeners, there was a significant two-way interaction effect for *accent* and *context* (F(4, 616) = 4.99, p = .00, PES = .03), and a significant main effect for *context* (F(2, 541) = 4.48, p = .01, PES = .02). However, there was no significant main effect for *accent* (F(2, 616) = .87, p = .42, PES = .00). Subsequent post-hoc comparisons (HSD) within the listener groups showed that the Audio Tour and Lecture aroused significantly higher *affect* than the Job Pitch (the same as the main effect in the overall analysis). The two-way interaction effect can be attributed to the different judgements in the German *listener group* of the British English accent for the three contexts: the Audio Tour achieved the lowest *affect* compared to the Job Pitch and Lecture.

For Singaporean listeners, there was a significant two-way interaction effect for *accent* and *context* (F(4, 541) = 3.79, p = .01, PES = .03), and main effects for *accent* (F(2, 541) = 6.67, p = .00, PES = .02) and *context* (F(2, 541) = 3.44, p = .03, PES = .01). Post-hoc comparisons (HSD) showed significantly higher *affect* for Dutch English (m = 3.55; SD = .79) compared to American English (m = 3.27; SD = .78) and British English (m = 3.30; SD = .83) (the same as the main effect in the overall analysis), and significantly lower *affect* for the Job Pitch (m = 3.24; SD = .93) compared to the Lecture (m = 3.41; SD = .67) but not the Audio Tour (m = 3.46; SD = .79). The two-way interaction effect for *accent* and *context* can be attributed to the different judgements of the three contexts within one *listener group*. There were no significant differences between the three contexts for Dutch English and American English.

For Spanish listeners, the two-way interaction effect for *accent* and *context* (F(4, 540) = 1.84, p = .12, PES = .01) was not significant. However, there were main interaction effects for *accent* (F(2, 540) = 9.66, p = .00, PES = .04) and *context* (F(2, 540) = 14.50, p = .00, PES = .01). Post-hoc comparisons (HSD) showed significantly lower *affect* for American English compared to Dutch English but not British English, and significantly lower *affect* for the Job Pitch compared to the Lecture and the Audio Tour (the same as the main effect in the overall analysis).

#### Dynamism

**Fig 7:**
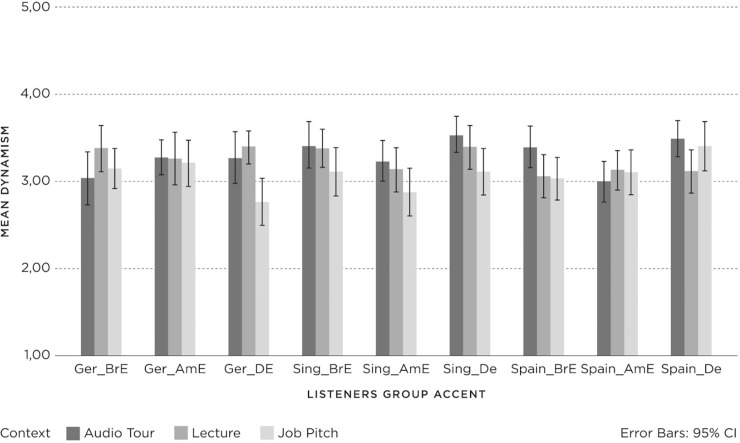
Dynamism means (1 = negative; 3 = neutral; 5 = positive) for Listener groups (Germany (Ger), Singapore (Sing), Spain,) per accent (British English (BrE), American English (AmE), Dutch English (DE)) and context (Audio Tour, Lecture, Job Pitch).

The results for *dynamism* in relation to *listener group*, *accent* and *context* are shown in Fig 7, with 95% confidence intervals.

Univariate analyses of variance showed that the three-way interaction between *listener group*, *accent*, and *context* (F(8, 1672) = 1.76, p = .08, PES = .01) and the two-way interaction between *accent* and *context* (F(4, 1672) = .70, p = .59, PES = .00) were not significant. However, the two-way interactions between, *accent* and *listener group* (F(4, 1672) = 2.59, p = .04, PES = .01), and *context* and *listener group* (F(4, 1672) = 3.00, p = .02, PES = .01) were significant. The interpretations of these interactions have to be seen in relation to their main effects, of which there was one for *context* (F(2, 1672) = 6.92, p = .00, PES = .01) on *dynamism*, but not for *listener group* (F(2, 1672) = .50, p = .61, PES = .00) and *accent* (F(2, 1672) = 2.90, p = .06, PES = .00). Post-hoc Tukey tests showed that the Job Pitch evoked significantly lower *dynamism* compared with the Audio Tour and the Lecture.

Subsequent univariate analyses were conducted within each *listener group* to investigate the effects of *accent* and *context* on *dynamism* per *listener group*. For German listeners, there was no significant two-way interaction effect for *accent* and *context* (F(4, 616) = 1.99, p = .10, PES = .01), and no main effect for *accent* (F(2, 541) = .53, p = .59, PES = .00). However, there was a main effect for *context* (F(2, 616) = 4.01, p = .02, PES = .01). Subsequent post-hoc comparisons (HSD) showed that, for the German listeners, the Job Pitch evoked significantly lower *dynamism* than the Lecture, but did not differ significantly from the Audio Tour. In addition, the German listeners who had indicated that Dutch-accented English was a non-native accent (m = 2.98; SD = 1.03) ascribed significantly lower *dynamism* to the speaker compared to listeners who indicated that Dutch-accented English was a native accent (m = 3.71; SD = .84) (F(1, 216) = 7.80, p = .01, PES = .04). Further testing showed that when the German listeners were able to recognize Dutch-accented English as coming from a Dutchman the assigned *dynamism* (m = 2.84; SD = .90) was significantly lower compared than when they believed the accent came from a native speaker of English (m = 3.71; SD = .84) (F(1, 216) = 6.86, p = .00, PES = .06).

For Singaporean listeners, there was no significant two-way interaction effect for *accent* and *context* (F(4, 541) = 1.97, p = .10, PES = .02), and no main effect for *context* (F(2, 541) = 2.00, p = .14, PES = .01). However, there was a main effect for *accent* (F(2, 541) = 3.68, p = .03, PES = .01). Post-hoc comparisons (HSD) showed that Dutch English evoked significantly higher *dynamism* than American English but not British English.

For the Spanish listeners, there was no significant two-way interaction effect for *accent* and *context* (F(4, 540) = .08, p = .99, PES = .00), but main effects for *accent* (F(2, 540) = 3.99, p = .02, PES = .02), and *context* (F(2, 540) = 6.85, p = .00, PES = .03). Post-hoc comparisons (HSD) showed significantly lower *dynamism* for American English (m = 3.08; SD = .97) compared to Dutch English (m = 3.35; SD = .94) but not British English (m = 3.31; SD = .98). For *context*, post-hoc comparisons (HSD) showed significantly lower *dynamism* for the Job Pitch compared to the Lecture and the Audio Tour, the same effect as we found in the overall analysis.

#### Summary table speaker evaluations

Table 4 provides an overview of the results for *speaker evaluations* for the three tested accents and contexts for all three listener groups.

**Table 4:** Summary results speaker evaluations (Status, Affect, Dynamism).

N = 1699	Status	Affect	Dynamism
**Listener Group (L)**	Spain > Germany	Spain, Singapore > Germany	NS
**Accent (A)**	NS	DE > BE, AE	NS
**Context (C)**	LE, AT > JP	LE, AT > JP	LE, AT > JP
**L x A**	Spain: DE, BE > AE	Spain: DE > AE Singapore: DE > BE, AE	Spain: DE > AE Singapore: DE > AE
**L x C**	Germany: LE, AT > JP Spain: LE, AT > JP Singapore: LE > JP	Germany: LE, AT > JP Spain: LE, AT > JP Singapore: LE > JP	Germany: LE > JP Spain: LE, AT > JP
**A x C**	NS	NS	NS
**L x A x C**	Spain BE: LE, AT > JP Spain DE: LE, AT > JP	Germany: BE: JP, LE > AT	NS

ᵃA = Accent; AE = American English; AT = Audio Tour; BE = British English; C = Context; DE = Dutch English; JP = Job Pitch; L = Listener group; LE = Lecture; NS = Not Significant.

## Discussion

### Speech understandability

Our first research question (RQ1) was aimed at investigating whether German, Spanish, and Singaporean listeners (*listener group*) displayed different *speech understandability* (*intelligibility*, *comprehensibility*, *interpretability*) in response to Dutch-accented English compared to standard British and American English accents (*accent*), and whether *context* (Lecture; Audio Tour; Job Pitch) affected their responses.

Our results show that *speech understandability* was not impacted overall by main effects of *accent* or *context* (see Table 2). However, the interaction effects (see again Table 2) showed that *listener groups* were affected differently by *accent* and *context* in their *speech understandability* responses. For the German listeners, standard American English was more *intelligible* compared to standard British English, and for the Spanish listeners, American English was more *intelligible* than Dutch English. These results might be explained by an increased exposure to American English for these two listener groups, however, there is no direct evidence to support this suggestion and we do not know why the Singaporean listeners did not show the same patters. *Context* was seen to affect *comprehensibility* and *interpretability* both within and between individual listener groups. Spanish listeners achieved higher *comprehensibility* of the audio tour than the German listeners, and higher *interpretability* of the audio tour than the German and Singaporean listeners.

For Spanish listeners, the content of the audio tour was interpreted best, followed by the lecture and job pitch. Overall, the results for the Spanish listeners illustrate that the audio tour was the context that was most understandable, in terms of *comprehensibilit*y and *interpretability*. The job pitch context evoked lower *comprehensibility* and *interpretability* within the three listener groups, and this confirms the findings from a similar study [28]. This particular response in the job pitch context might be explained by a lack of familiarity with such a context, as our listeners were not selected specifically on the basis of experience with job pitches for retail management positions.

We had expected (expectation 1a) that Spanish listeners would display a lower level of *speech understandability* of the three accents compared to German and Singaporean listeners. This expectation was confirmed for *intelligibility*. Expectation 1a was also confirmed for Spanish listeners compared to German listeners in terms of the *interpretability* of the job pitch. On the one hand, these results might be an illustration of previously reported weaker language skills by Spanish listeners compared to German and Singaporean listeners [44]. On the other, they might be explained by language distance, as Spanish listeners’ L1, Spanish is not part of the West Germanic language family as German, Dutch, and English. It might be the case that due to greater language distance between for example Spanish and English speakers, compared to German and English speakers, it is more difficult for Spanish speakers of English to achieve a certain level of English language fluency. In contrast, and perhaps surprisingly, in the audio tour context, Spanish listeners achieved higher *comprehensibility* and *interpretability* compared to German listeners. This might imply that despite Spanish listeners’ self-reported weaker fluency and their lower general *intelligibility* scores compared to German and Singaporean listeners, they were still able to correctly comprehend and interpret a particular communication context, in our case an audio tour. In turn, this might imply that an L2 listener’s English fluency need not be very high in this context for them to correctly comprehend and interpret L2 speech. In addition, these findings indicate that *comprehensibility* and *interpretability* of speech can be relatively high even though *intelligibility* is relatively low, which might imply that correctly distinguishing every word (*intelligibility*) is not a prerequisite for understanding speech content (*comprehensibility*) and the communicative purpose of that content (*interpretability*). Thus, distinguishing between these three factors of *speech understandability* in accentedness research is worthwhile, as this approach can generate more nuanced findings, and can throw light on the relationship between–and the assumed cumulative stages in–understandability of (accented) speech.

In terms of Singaporean listeners, we had expected (expectation 1b) that they would display higher *speech understandability* than German and Spanish listeners. This expectation was not confirmed for German listeners, but it was confirmed for Spanish listeners with respect to *intelligibility*. The assumed advantage for Singaporean listeners based on their higher English language skills and assumed greater exposure to various English varieties only partly emerged from the data. This might indicate that having a high level of English combined with potential exposure to varieties of English does not automatically lead to better understanding of L1 and L2 English accents.

In the context of a general discussion on the existence of an LFE speech community versus traditional SLA language learning perspectives, our results indicate that effective language learning and language use may not necessarily require ‘mastering the ‘target’ language’ (L1 language) at a high level and being exposed to different varieties of that language.

Our last expectation regarding understandability (*expectation 1c*) was that the lecture context would evoke higher *speech understandability* compared to the job pitch and audio tour communication contexts. Our assumption was that our highly educated listeners would be most familiar with the lecture context, having followed higher or tertiary education. By extension, we posited that *speech understandability* would also be higher in the lecture context because of this familiarity compared to the other two contexts. *Expectation 1c* was confirmed only partly, as different patterns emerged for the different listener groups and with respect to the individual components of *speech understandability*. For instance, for *intelligibility* our expectation was only confirmed within the Spanish listener group. For *comprehensibility* it was confirmed for German listeners. For Singaporean listeners the lecture context only evoked higher *comprehensibility* compared to the job pitch (but not the audio tour).

Finally, with respect to *interpretability*, both Spanish and Singaporean listeners showed higher *interpretability* of the lecture compared to the job pitch, but not the audio tour. Overall, when the lecture evoked higher *speech understandability* compared to other contexts this was only the case within the listener groups and almost only compared to the job pitch. Interestingly, our listeners did not confirm our expectation for the *speech understandability* of the lecture compared to the audio tour, which often achieved equal or higher *speech understandability* compared to the lecture. The audio tour speech sample focused on explaining the type of art that was on display in an art gallery (e.g. Australian aborigine art; photography collections from specific time periods) and was selected from an internationally acknowledged official English certificate listening exam to reliably assess *speech understandability*. It might have been the case that our assumption with respect to the familiarity of our listeners with the three contexts was incorrect and that our listeners were familiar with an audio tour.

The results make clear that context affects *speech understandability* in various ways. It is important to understand in more detail what factors might play a role in this context effect, and therefore, further research on this topic is required. It is important to emphasize that overall understandability scores of our respondents were very high. Lack of understanding was not a constraint for the respondents in evaluating our speakers.

### Speaker evaluations

Our second research question (RQ2) was aimed at investigating whether German, Spanish, and Singaporean listeners (*listener group*) displayed different *speaker evaluations* in response to Dutch-accented English compared to standard British and American English accents (*accent*), and whether *context* (Lecture; Audio Tour; Job Pitch) affected their responses.

Our results show that Dutch-accented English does not have negative effects on a speaker’s *status*, *affect*, and *dynamism* (*speaker evaluations*) compared with standard British and American English. In fact, it even evokes higher *affect* compared to both L1 English accents (see Table 4). However, *listener group* did impact *speaker evaluations* for *status* and *affect*, with Spanish listeners evaluating all speakers, regardless of accent, as having higher *status* compared to German listeners, and both Singaporean and Spanish listeners assigning higher *affect* to all speakers, regardless of accent, compared to German listeners. Finally, *context* affected *speaker evaluations* in that the job pitch evoked lower speaker evaluations compared to the lecture and the audio tour.

We had expected (expectation 2a) that German and Spanish listeners would ascribe Dutch-accented English lower *status* compared to standard British and American English accents, but this was not the case. German listeners’ *speaker evaluations* were not affected by *accent*, and Spanish listeners assigned Dutch-accented English speakers significantly more positive *status*, *affect*, and *dynamism* than American English speakers. This suggests that in communications between L2 English speakers, having an L2 English accent can evoke equal or even higher *speaker evaluations* than a native English accent. In addition, our listeners’ responses confirm Canagarajah’s [11] assertion that the LFE speech community members will likely possess flexible language attitudes, which in turn suggests that they do not hold the traditional SLA perspective on what makes for an effective L2 English speaker.

Our second expectation with regard to *speaker evaluations* (expectation 2b) was that Singaporean listeners would not respond differently to Dutch-accented English compared to standard British and American English accents. This was indeed found to be the case for *status*. Against expectations, however, for Singaporean listeners, Dutch-accented English aroused higher *affect* and *dynamism* than standard American English, and Dutch-accented English aroused higher *affect* than standard British English. This might indicate that, to Singaporeans, having an L2 accent does not negatively affect judgements of a speaker’s abilities. This, in turn, might stem from living in a multicultural and multilingual society in which accent variety is the norm, and in which being an L2 English speaker can actually be an advantage if the intention is to be liked (*affect*) or perceived as dynamic (*dynamism*). In other words, this might again be an indication that the traditional SLA perspective does not automatically apply. It also illustrates the acceptance of L2 English within a specific LFE speech community, in this case Singapore.

The third expectation (expectation 2c) was that the job pitch context would evoke lower *speaker evaluations* compared to the lecture and audio tour context. This expectation was confirmed, because for all three listener groups the *status*, *affect* and *dynamism* evoked by speakers in the job pitch context was lower compared to that assigned to speakers in the lecture and audio tour contexts. German listeners assigned speakers in the lecture context higher *status*, *affect*, and *dynamism* compared to the job pitch. They also assigned speakers in the audio tour context higher *status* and *affect* compared to speakers in the job pitch context. Spanish listeners ascribed higher *status*, *affect*, and *dynamism* to speakers in the lecture and the audio tour contexts compared to the speakers in the job pitch context. Finally, Singaporean listeners ascribed speakers in the lecture context higher *status* and *affect* compared to the speakers in the job pitch context. These findings illustrate that, except for Singaporeans and *dynamism*, the job pitch arouses lower *speaker evaluations*. Interestingly, the German listeners who evaluated the standard British English accent assigned higher *affect* to speakers in the job pitch and audio tour contexts compared to speakers in the lecture context, which is in contrast with the general finding that German listeners assigned speakers in the lecture and audio tour contexts higher *affect* compared to the speakers in the job pitch context. This suggests that listeners may hold specific expectations of speakers in different communication contexts, and that when these expectations are violated [69, 70], listeners evaluate speakers more negatively. To what extent listener expectations with regard to accentedness in different contexts actually play a role in their perceptions of speakers deserves further investigation.

Our findings do not concur with previous research that found that both L1 and L2 speakers of English tend to assign L2 English accents lower *status* compared to L1 English accents [e.g. 23, 18–34, 68]. In the present study, there was a clear tolerance of L2 English speakers as listeners towards L2 English accent, as also indicated by a smaller number of studies [e.g. 14, 28]. Our results show that L2 English speakers assign L1 and L2 English accents equal *status* (e.g. Singapore, Germany), and even assign an L2 English accent higher *status* (Spain) than an L1 English accent. In addition, an L2 English accent has been shown to actually evoke higher *affect* (Spain, Singapore) and *dynamism* (Spain, Singapore) compared to L1 English accents [confirmed by 28, 87].

During the course of our experiment we realized that listeners who had correctly identified the accents as native or non-native might have different responses than those who had not. We assessed this effect on the basis of the listeners’ answers to the question on the nationality of the speaker. Our findings showed that in general there were no significant differences between listeners who had correctly identified the accents and the listeners who had incorrectly identified the accents. This suggests that familiarity with an L2 English accent does not necessarily lead to better speech understanding nor more positive or negative speaker evaluations. This might lend support to the notion that an LFE speech community might exist in which unfamiliar L1 and L2 English accents are relatively easily absorbed, understood and not necessarily evaluated negatively or positively. However, the German listeners who had identified the Dutch-accented English as non-native English, ascribed lower *status* and *dynamism* to the speaker than the German listeners who stated the Dutch-accented English was native English. This suggests that these German listeners do adhere to the traditional SLA norm. In addition, Spanish listeners who believed the standard British English accent was non-native in the job pitch context ascribed higher *status* to the speaker compared to listeners who had indicated that the British English accent was native. This shows again that Spanish listeners preferred the L2 over an L1 English accent, and that they do not adhere to the traditional SLA norm, even in a very specific context. These effects were not part of the main questions and expectations of this study, but yielded interesting results nonetheless that call for further research.

### Practical implications

In general, in an LFE speech community setting, *speech understandability* and *speaker evaluations* are not negatively impacted by Dutch-accented English, but are also not positively affected by standard British or American English accents. This means that the traditional SLA perspective no longer applies to an LFE setting, where flexible language norms might hold instead. Most ‘foreign’ or L2 language education is aimed at training language learners to become fluent on a ‘native level’, in various degrees, in a ‘target’ language, which typically is a standard, L1 variety of the ‘target’ language. In the case of English, L2 English speakers and English language learners now outnumber L1 English speakers [12]. Our results suggest that educators and learners of English might need to review their perceptions of what it means to be a successful learner and speaker of English, and potentially rethink educational practices. Our results show, contrary to many accentedness studies, that an L2 English accent within the LFE speech community does not have to hinder effective communication. First, an L2 English accent can be as understandable as standard L1 English accents, even if listeners are not familiar with that particular L2 English accent, which is a realistic scenario in international interactions. Second, if the intent is to evoke evaluations of high status, affect, and dynamism in listeners in the LFE speech community, being an L2 English speaker with an L2 English accent can actually be more beneficial compared to having a L1 English accent. This means that for L2 English speakers accent training aimed at achieving a native-like accent may not be necessary if achieving understandability and positive speaker evaluations are the main learning objectives.

Context, however, did impact *speech understandability* within listener groups and *speaker evaluations* in general and within listener groups with the job pitch context often achieving lower understanding and lower evaluations compared to the lecture and audio tour contexts. These results suggests that listeners might not necessarily respond to a speaker’s accent, instead, they react to communication context, topic and content, which might be connected to specific communication expectations. As a result, breakdowns in communication between speakers of English with various L1 backgrounds should not solely be evaluated in terms of speakers’ language skills and accentedness, but should also be analyzed in relation to the familiarity and requirements of the communication context and content.

### Limitations and future research

This study has a number of limitations. First, we used only one male matched-guise speaker in our experiment. This could have led to reactions that represent a response to this particular male speaker, and to male speakers only. At the same time, the use of the matched-guise technique contributed to the validity of our results, since they cannot be attributed to the voice characteristics of individual speakers (as might have been the case had verbal guises been used).

A second limitation was that the listeners were selected to represent people who in general would be most likely to be familiar with the three selected contexts. With hindsight, however, professionals who regularly interact in these contexts, such as HR managers in the job pitch context, would have been the optimal choice, but this was not possible due to practical reasons. Also, the three communication contexts concerned different topics and content. The topic of marketing featured in the lecture context, aboriginal art in the audio tour, and the position of retail manager in the job pitch. The content featured in each context was the definition of marketing in the lecture, the work at display in the aboriginal art exhibition in the audio tour, and the achievements of the interviewee for the position of retail manager in the job pitch. In general, the three contexts were understood equally well, implying that the associated topics and content did not impact the listeners’ understanding of the speakers either. However, there were differences within listener groups between contexts for *speech understandability* and there were significantly different responses to the contexts for *speaker evaluations* in general and within listener groups, especially for the job pitch context. These differences might have been caused not only by communication context but also by the topic and/or content in a particular context. All listeners had to evaluate all three contexts in the same order, and as a result, the topic and content of each context could not be too similar since this might have impacted listeners’ responses, for instance in terms of *speech understandability*. It is recommended therefore that future accentedness research should investigate the role of listeners’ familiarity with specific communication contexts and to what extent variations in topic and content within a particular communication context impact listeners’ responses to accented speech.

A third limitation is that the accents studied here all come from a West Germanic language. It is recommended that future studies should select accents of typologically diverse language families to determine which accent properties, including specific phonemic characteristics or other linguistic features, such as prosody, affect listeners’ responses, and to what extent they do so.

In terms of the assessment of *speech understandability* based in Kachru and Smith’s [67] three speech understanding components, *intelligibility*, *comprehensibility*, and *interpretability* yielded results that provide useful insights into the levels at which a varied group of listeners are able to understand speech. However, in order to assess *comprehensibility*, and *interpretability*, two statements on each speech sample were presented to listeners on its content (*comprehensibility*) and its communicative purpose (*interpretability*), which had to be judged as correct or incorrect. This is a very general manner to assess these components of *speech understandability*. Future research should seek more detailed measurements to further understand how these components can be studied best, and also assess how these three components are correlated, with the aim to understand how *speech understandability* works and how better understanding can be achieved.

Additionally, in terms of *speaker evaluations*, the listeners groups displayed clear consensus with respect to *status* and similarly so for *affect*, which provides further support for the cross-cultural agreement that exist between listeners in determining someone’s social status and their likeability. This was not the case for *dynamism*. The *dynamism* questionnaire items loaded as part of a third separate factor for the German and Spanish listeners, but did not for the Singaporean listeners. For Singaporeans, two of three dynamism items were considered part of *affect* and not *dynamism*, and thus these concepts might have similar meaning to Singaporeans. However, for German and Spanish listeners one of the three *dynamism* items ‘energetic’ was considered separate from *status* and *affect*, and dynamism does appear to represent an important separate aspect of someone’s personality. In order to be able to compare the responses to this *dynamism* item for all listener groups, which was a main aim of this study, *dynamism* was defined as representing a listeners’ perceptions of a speaker’s energy. Further cross-cultural research on how different listener groups define a speaker’s dynamism could help researchers select items that optimally study the perceptions listeners have of different speaker groups.

The speaker evaluations were carried out on the basis of explicit speaker evaluation measurements (status, affect, dynamism item responses), which is traditionally the most commonly used speech evaluation research method. Relatively recently, implicit speaker evaluation assessments have been conducted which have yielded promising results, as shown by Pantos and Perkins [89, 90]. They observed more negative speaker evaluations with implicit (Implicit Association Test) speaker evaluation measurements compared with explicit speaker evaluation measurements. Future research might gain more understanding of the different types of speaker evaluations by listeners by incorporating both explicit and implicit assessments of speaker evaluations.

Finally, the data was collected via Qualtrics, an online data collection service, which is increasingly used by scientists across the globe. Qualtrics guarantees high quality data collection and it was the only way that allowed swift international data collection of a significant number of responses. We do not see this as a limitation, but as an incentive to supplement our data with accent evaluation research on a more restricted local level, with alternative ways of sampling of respondents.

## Supporting information

S1 File Speech sample links: Matched guises, filler, controls. (PDF)

S2 File Speaker evaluations and speech understandability questionnaire questions Germany. (PDF)

S3 File Speaker evaluations and speech understandability questionnaire questions Spain. (PDF)

S4 File Speaker evaluations and speech understandability questionnaire questions Singapore. (PDF)

S5 File Link SPSS data. (PDF)

S1 Table Listener groups (N = 540; n = 30) and speech sample design for each country (Germany, Singapore, Spain) per accent (BrE = standard British English; AmE = standard American English or DE = Dutch-accented English) and context (Lecture, Audio Tour, Job Pitch). (PDF)

S2 Table Country of origin of speakers estimates per listener group and per accent in rounded %. (PDF)

S3 Table Germany speech understandability and speaker evaluations per accent (British English, American English, Dutch English; 1 = negative; 3 = neutral; 5 = positive) and context (Lecture, Audio Tour, Job Pitch). ᵃmax. 11 words; ᵇmax. 12 words intelligible. N = 617; n = number of listeners per accent and context.
(PDF)

S4 Table Singapore speech understandability and speaker evaluations per accent (British English, American English, Dutch English; 1 = negative; 3 = neutral; 5 = positive) and context (Lecture, Audio Tour, Job Pitch). ᵃmax. 11 words; ᵇmax. 12 words intelligible. N = 542; n = number of listeners per accent and context.
(PDF)

S5 Table Spain speech understandability and speaker evaluations per accent (British English, American English, Dutch English; 1 = negative; 3 = neutral; 5 = positive) and context (Lecture, Audio Tour, Job Pitch). ᵃmax. 11 words; ᵇ max. 12 words intelligible. N = 540; n = number of listeners per accent and context.
(PDF)
