# Supplementary material for: Where does a ‘foreign’ accent matter? German, Spanish and Singaporean listeners’ reactions to Dutch-accented English, and standard British and American English accents
Source: PLoS One. 2020 Apr 29;15(4):e0231089. doi: 10.1371/journal.pone.0231089 (PMC7190091; doi:10.1371/journal.pone.0231089)
Supplement: S1 File — (PDF) [file pone.0231089.s001.pdf]

S1 File. Speech sample links: matched guises, filler, controls.

The speech sample links below redirect to one speech sample per accent (standard British, standard American English, Dutch English) and communication context (lecture, audio tour, job pitch), per speaker (matched-guise speaker; filler speaker; control speakers). For the controls, three speech sample links are available as examples (for each accent one).

Matched-guise speaker Lecture

- standard British English https://cls.ru.nl/webexp-media/HV_BE_L.html
- standard American English https://cls.ru.nl/webexp-media/HV_AE_L.html
- Dutch English https://cls.ru.nl/webexp-media/HV_DE_L.html

Lecture speech sample text:

Last week we looked at some general principles associated with marketing and today I'd like to look at some of those points in a little more detail. So what is marketing? Or to put it another way, what does the term marketing mean? Many people think of it simply as the process of selling and advertising. And this is hardly surprising when every day we are bombarded with telephone sales. But selling and advertising are only two functions of marketing. In fact, marketing, more than any other business function, deals with customers. So perhaps the simplest definition is this: marketing is the delivery of customer value and satisfaction at a profit. In other words, finding customers, keeping those customers happy and making money out of the process.

Matched-guise speaker Audio tour

- standard British English https://cls.ru.nl/webexp-media/HV_BE_A.html
- standard American English https://cls.ru.nl/webexp-media/HV_AE_A.html
- Dutch English https://cls.ru.nl/webexp-media/HV_DE_A.html

Audio tour speech sample text:

Welcome to the City Art Gallery and to our gallery audio tour. The gallery was first established in 1875 and is now one of the city's most popular attractions. The building has undergone a number of changes over the years, the most recent addition being the extension to the ground floor, which was opened in 1988. The Gallery houses some of the finest works of art in Australia, including Aboriginal, European and Asian paintings. There is a

comprehensive Australian collection, which includes works from the early colonial period to the present day. In addition to the paintings on display, we have an excellent collection of photography, with photographs dating from the nineteenth century. Now let's begin our tour in the nineteenth century Australian room.

Matched-guise speaker Job pitch

- standard British English https://cls.ru.nl/webexp-media/HV_BE_J.html
- standard American English https://cls.ru.nl/webexp-media/HV_AE_J.html
- Dutch English https://cls.ru.nl/webexp-media/HV_DE_J.html

Job Pitch speech sample text:

I'm a seasoned Retail Manager strong in developing training programs and loss prevention techniques that have resulted in revenue savings of over 2.3 Million during the past 11 years. I would say that my greatest strength is my ability to follow through. I have found that I am most successful when I pay attention to every piece of the product cycle, from the first contact, to the thank you at the completion of the project. My greatest weakness is my tendency to overthink a situation. I sometimes take too much time to strategize on a sale, and find in the end that my initial plan was the one that was the best.

Filler speaker

- standard British English https://cls.ru.nl/webexp-media/CG_BE_F.html

Examples of controls or native speakers for each accent (in the lecture context only)

- standard British English https://cls.ru.nl/webexp-media/JC_BE_L.html
- standard American English https://cls.ru.nl/webexp-media/PG_AE_L.html
- Dutch English https://cls.ru.nl/webexp-media/CJ_DE_L.html
