# Supplementary material for: Where does a ‘foreign’ accent matter? German, Spanish and Singaporean listeners’ reactions to Dutch-accented English, and standard British and American English accents
Source: PLoS One. 2020 Apr 29;15(4):e0231089. doi: 10.1371/journal.pone.0231089 (PMC7190091; doi:10.1371/journal.pone.0231089)

S4 File. Speaker evaluations and speech understandability questionnaire questions Germany

Speaker evaluations question screenshot, job pitch context.

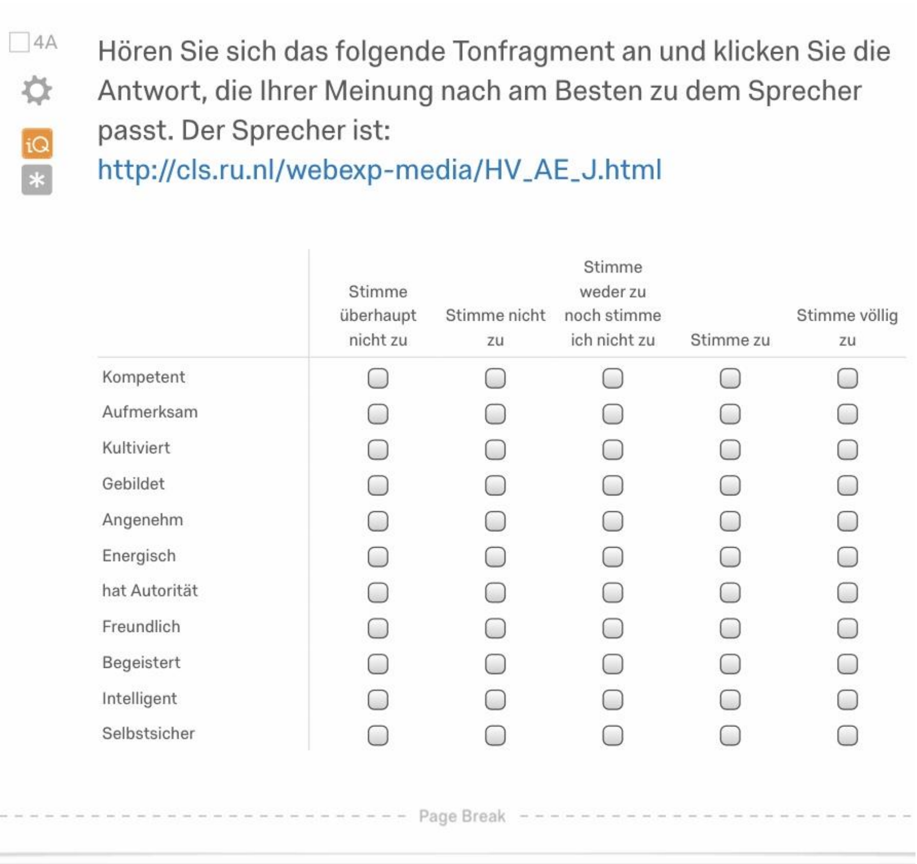

4A

Hören Sie sich das folgende Tonfragment an und klicken Sie die Antwort, die Ihrer Meinung nach am Besten zu dem Sprecher passt. Der Sprecher ist: http://cls.ru.nl/webexp-media/HV_AE_J.html

	Stimme überhaupt nicht zu	Stimme nicht zu	Stimme weder zu noch stimme ich nicht zu	Stimme zu	Stimme völlig zu
Kompetent					
Aufmerksam					
Kultiviert					
Gebildet					
Angenehm					
Energisch					
hat Autorität					
Freundlich					
Begeistert					
Intelligent					
Selbstsicher					

Page Break

Speech understandability questions screenshot (interpretability followed by comprehensibility), job pitch context.

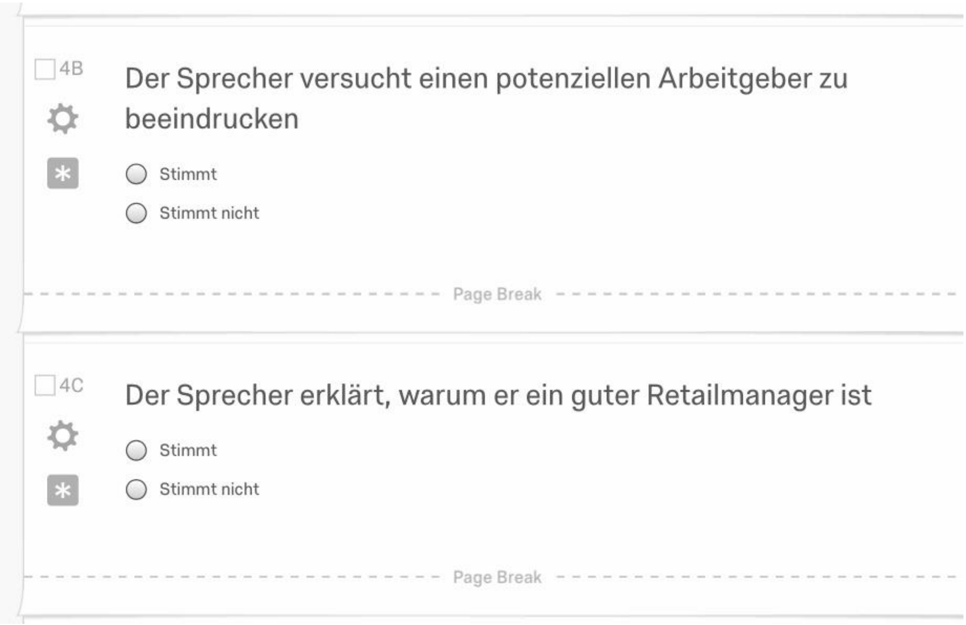

4B

Der Sprecher versucht einen potenziellen Arbeitgeber zu beeindrucken

- Stimmt
- Stimmt nicht

Page Break

4C

Der Sprecher erklärt, warum er ein guter Retailmanager ist

- Stimmt
- Stimmt nicht

Page Break

Speech understandability question screenshot (intelligibility), job pitch context.

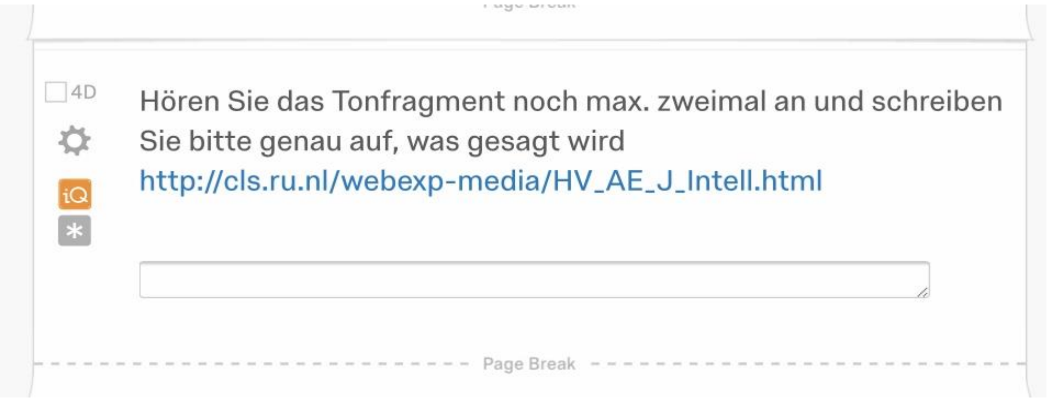

Supplement: S2 File — (PDF) [file pone.0231089.s002.pdf]
