# Supplementary material for: Where does a ‘foreign’ accent matter? German, Spanish and Singaporean listeners’ reactions to Dutch-accented English, and standard British and American English accents
Source: PLoS One. 2020 Apr 29;15(4):e0231089. doi: 10.1371/journal.pone.0231089 (PMC7190091; doi:10.1371/journal.pone.0231089)

S5 File. Speaker evaluations and speech understandability questionnaire questions Spain

Speaker evaluations question screenshot, audio tour context.

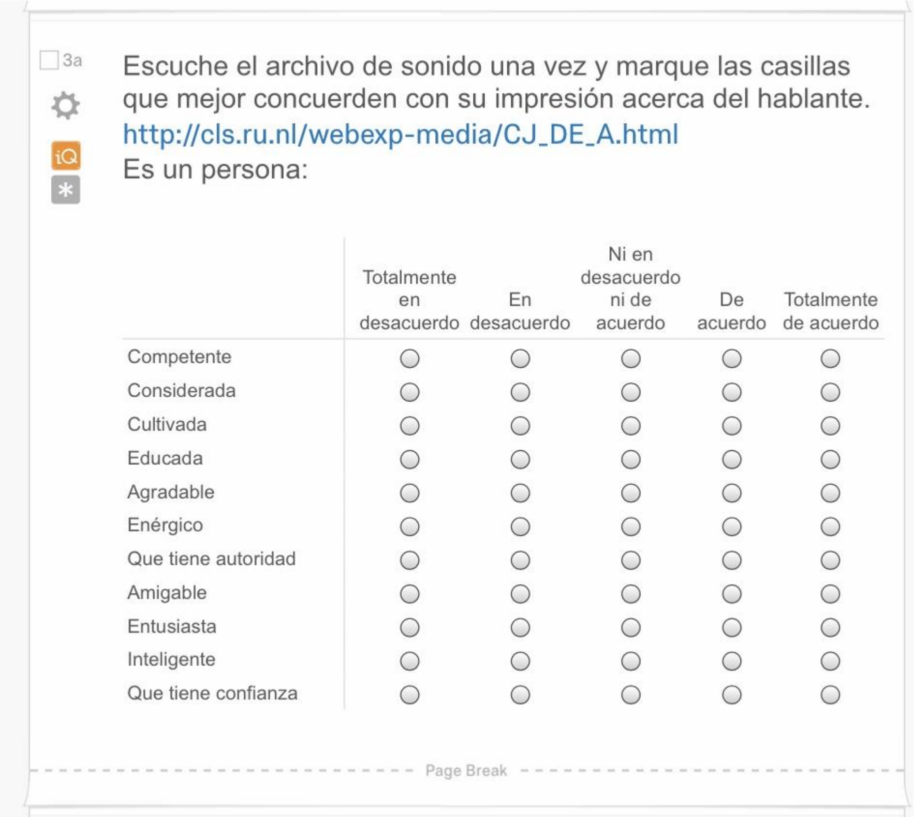

3a

Escuche el archivo de sonido una vez y marque las casillas que mejor concuerden con su impresión acerca del hablante.
http://cls.ru.nl/webexp-media/CJ_DE_A.html
Es un persona:

	Totalmente en desacuerdo	En desacuerdo	Ni en desacuerdo ni de acuerdo	De acuerdo	Totalmente de acuerdo
Competente	○	○	○	○	○
Considerada	○	○	○	○	○
Cultivada	○	○	○	○	○
Educada	○	○	○	○	○
Agradable	○	○	○	○	○
Enérgico	○	○	○	○	○
Que tiene autoridad	○	○	○	○	○
Amigable	○	○	○	○	○
Entusiasta	○	○	○	○	○
Inteligente	○	○	○	○	○
Que tiene confianza	○	○	○	○	○

Page Break

Speech understandability questions screenshot (interpretability followed by comprehensibility), audio tour context.

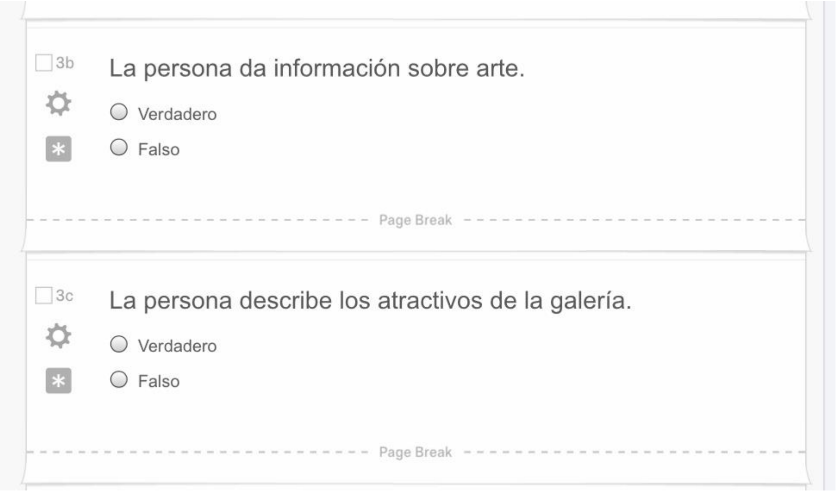

3b

La persona da información sobre arte.

○ Verdadero
○ Falso

Page Break

3c

La persona describe los atractivos de la galería.

○ Verdadero
○ Falso

Page Break

Speech understandability question screenshot (intelligibility), audio tour context.

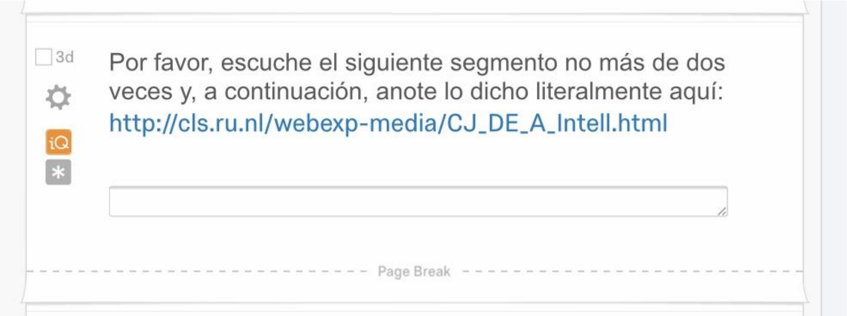

Supplement: S3 File — (PDF) [file pone.0231089.s003.pdf]
