# Supplementary material for: Where does a ‘foreign’ accent matter? German, Spanish and Singaporean listeners’ reactions to Dutch-accented English, and standard British and American English accents
Source: PLoS One. 2020 Apr 29;15(4):e0231089. doi: 10.1371/journal.pone.0231089 (PMC7190091; doi:10.1371/journal.pone.0231089)

S6 File. Speaker evaluations and speech understandability questionnaire questions Singapore

Speaker evaluations question screenshot, audio tour context.

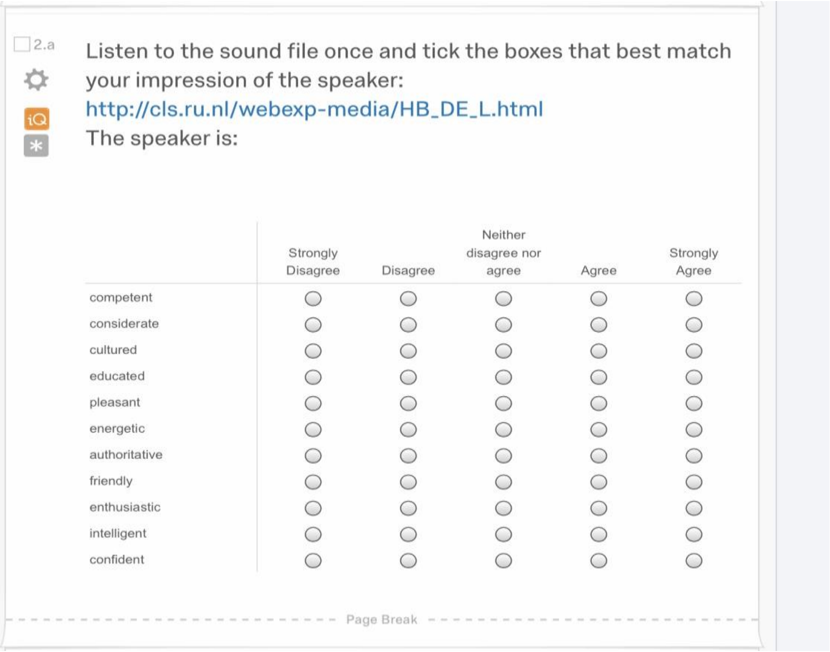

2.a

Listen to the sound file once and tick the boxes that best match your impression of the speaker:
http://cls.ru.nl/webexp-media/HB_DE_L.html
The speaker is:

	Strongly Disagree	Disagree	Neither disagree nor agree	Agree	Strongly Agree
competent	○	○	○	○	○
considerate	○	○	○	○	○
cultured	○	○	○	○	○
educated	○	○	○	○	○
pleasant	○	○	○	○	○
energetic	○	○	○	○	○
authoritative	○	○	○	○	○
friendly	○	○	○	○	○
enthusiastic	○	○	○	○	○
intelligent	○	○	○	○	○
confident	○	○	○	○	○

Page Break

Speech understandability questions screenshot (interpretability followed by comprehensibility), lecture context.

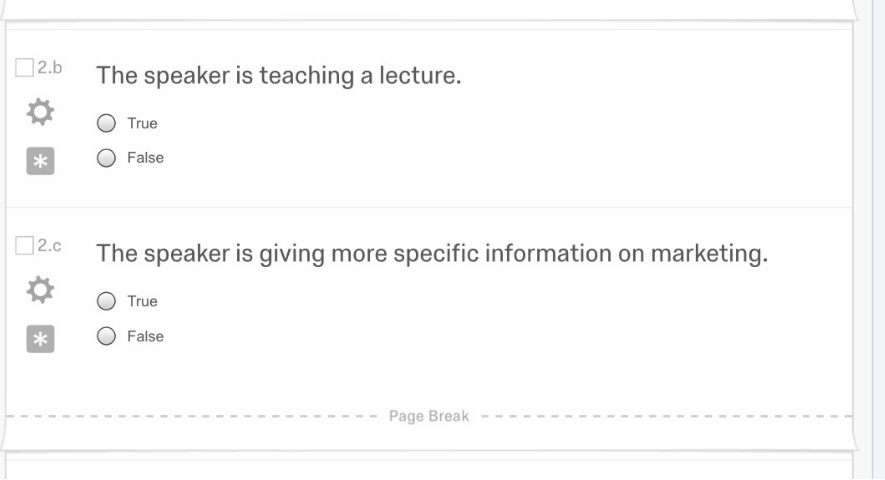

2.b

The speaker is teaching a lecture.

○ True

○ False

2.c

The speaker is giving more specific information on marketing.

○ True

○ False

Page Break

Speech understandability question screenshot (intelligibility), lecture context.

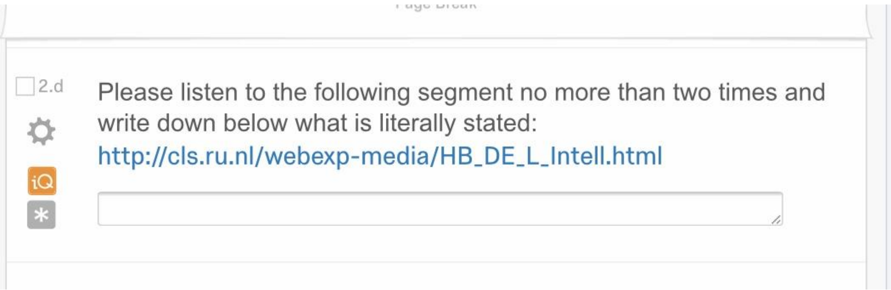

Supplement: S4 File — (PDF) [file pone.0231089.s004.pdf]
