# Supplementary material for: Where does a ‘foreign’ accent matter? German, Spanish and Singaporean listeners’ reactions to Dutch-accented English, and standard British and American English accents
Source: PLoS One. 2020 Apr 29;15(4):e0231089. doi: 10.1371/journal.pone.0231089 (PMC7190091; doi:10.1371/journal.pone.0231089)
Supplement: S5 File — (PDF) [file pone.0231089.s005.pdf]

S7 Data file

Please click on this OSF link to gain access to the data file:

https://osf.io/7c9yh/?view_only=9933c341f23446aeaec3e199e2725ef2
