# Supplementary material for: Where does a ‘foreign’ accent matter? German, Spanish and Singaporean listeners’ reactions to Dutch-accented English, and standard British and American English accents
Source: PLoS One. 2020 Apr 29;15(4):e0231089. doi: 10.1371/journal.pone.0231089 (PMC7190091; doi:10.1371/journal.pone.0231089)
Supplement: S1 Table — (PDF) [file pone.0231089.s006.pdf]

S2 Table. Listener groups (N=540; n=30) and speech sample design for each country (Germany, Singapore, Spain) per accent (BrE = standard British English; AmE = standard American English or DE = Dutch-accented English) and context (Lecture, Audio Tour, Job Pitch).

Listener groups	Speech sample order	Listener groups	Speech sample order
1.	*Filler BrE<br>**MG BrE Lecture<br>***C1-NS AmE Audio Tour<br>C1-NS BrE Job Pitch	10.	Filler BrE<br>MG AmE Lecture<br>C2-NS AmE Audio Tour<br>C2-NS BrE Job Pitch
2.	Filler BrE<br>C1-NS DE Lecture<br>MG BrE Audio Tour<br>C1-NS AmE Job Pitch	11.	Filler BrE<br>C2-NS DE Lecture<br>MG AmE Audio Tour<br>C2-NS AmE Job Pitch
3.	Filler BrE<br>C1-NS AmE Lecture<br>C1-NS DE Audio Tour<br>MG BrE Job Pitch	12.	Filler BrE<br>C2-NS AmE Lecture<br>C2-NS DE Audio Tour<br>MG AmE Job Pitch
4.	Filler BrE<br>MG AmE Lecture<br>C2-NS AmE Audio Tour<br>C1-NS DE Job Pitch	13.	Filler BrE<br>MG DE Lecture<br>C1-NS AmE Audio Tour<br>C2-NS DE Job Pitch
5.	Filler BrE<br>C1-NS BrE Lecture<br>MG AmE Audio Tour<br>C2-NS AmE Job Pitch	14.	Filler BrE<br>C2-NS BrE Lecture<br>MG DE Audio Tour<br>C1-NS AmE Job Pitch
6.	Filler BrE<br>C2-NS AmE Lecture<br>C1-NS BrE Audio Tour<br>MG AmE Job Pitch	15.	Filler BrE<br>C1-NS AmE Lecture<br>C2-NS BrE Audio Tour<br>MG DE Job Pitch
7.	Filler BrE<br>MG DE Lecture<br>C2-NS DE Audio Tour<br>C2-NS BrE Job Pitch	16.	Filler BrE<br>MG BrE Lecture<br>C1-NS DE Audio Tour<br>C1-NS BrE Job Pitch
8.	Filler BrE<br>C2-NS BrE Lecture 1<br>MG DE Audio Tour<br>C2-NS DE Job Pitch	17.	Filler BrE<br>C1-NS BrE Lecture<br>MG BrE Audio Tour<br>C1-NS DE Job Pitch
9.	Filler BrE<br>C2-NS DE Lecture<br>C2-NS BrE Audio Tour<br>MG DE Job Pitch	18.	Filler BrE<br>C1-NS DE Lecture<br>C1-NS BrE Audio Tour<br>MG BrE Job Pitch

*Filler BrE = Filler speaker with British English accent; **MG = matched-guise speaker; ***C1 or C2 = control speaker 1 or 2.
