# Supplementary material for: Where does a ‘foreign’ accent matter? German, Spanish and Singaporean listeners’ reactions to Dutch-accented English, and standard British and American English accents
Source: PLoS One. 2020 Apr 29;15(4):e0231089. doi: 10.1371/journal.pone.0231089 (PMC7190091; doi:10.1371/journal.pone.0231089)
Supplement: S2 Table — (PDF) [file pone.0231089.s007.pdf]

S3 Table. Country of origin of speakers estimates per listener group and per accent in rounded %

	**Standard British English**	**Standard American English**	**Dutch-accented English**
**Germany (n=617)**	47.4% correct<br><br>Incorrect:<br>31.4% U.S.;<br>13.7% Australia / New Zealand;<br>4.0% (Western) Europe/ L2; 2.3% German; 1.1% Africa	47.5% correct<br><br>Incorrect:<br>31.9% U.K.;<br>12.7% Australia / New Zealand<br>4.9% (Western) Europe/ L2; 2.5% German; 0.5% Africa	40.1% correct<br><br>Incorrect:<br>19.8% U.K.;<br>24.0% Europe, France, Belgium/ L2/ the West;<br>6.0% U.S.; 6.0% Germany;<br>4.1% Asia; 0.5% Australia / New Zealand
**Spain (n=540)**	57.4% correct<br><br>Incorrect:<br>22.8% U.S.;<br>14.2% Australia / New Zealand; 3.6% Europe/ German/ L2;<br>0.6%Netherlands;<br>0.6% South America;<br>0.6%Spain	42.3% correct<br><br>Incorrect:<br>36.2% U.K.;<br>14.1% Australia / New Zealand; 5.0% Europe/ German/ L2;<br>0.6% Netherlands;<br>0.6% Asia; 0.6% Spain; 0.6% South America	1.7% correct;<br><br>Incorrect:<br>35.6% Europe, France, Germany, Russia, Belgium/ L2; 28.2% U.K.; 16.7 % U.S.; 11.5% Australia/ New Zealand; 4.0% Asia; 1.7% Spain; 0.6% Africa.
**Singapore (n=542)**	41.0% correct<br><br>Incorrect:<br>28.1% U.S.;<br>17.5% Australia / New Zealand<br>7.9% Europe<br>2.8% Asia<br>2.2% Netherlands;<br>0.5% L2	48.5% correct<br><br>Incorrect:<br>23.4% U.K.;<br>15.0% Australia / New Zealand;<br>8.4% Europe; 2.4% Asia; 0.6% Netherlands; 1.2%	1.7% correct<br><br>Incorrect:<br>54.6% Europe, France, Germany, Russia, Italy;<br>13.2% U.K.; 11.5 % U.S.;<br>9.2% Singapore/Asia; 6.9% Australia / New Zealand;<br>2.2% the West/ western countries/ L2; 0.6% Africa

		South America; 0.5% Africa	
