# Supplementary material for: Where does a ‘foreign’ accent matter? German, Spanish and Singaporean listeners’ reactions to Dutch-accented English, and standard British and American English accents
Source: PLoS One. 2020 Apr 29;15(4):e0231089. doi: 10.1371/journal.pone.0231089 (PMC7190091; doi:10.1371/journal.pone.0231089)
Supplement: S3 Table — ᵃmax. 11 words; ᵇmax. 12 words intelligible. N = 617; n = number of listeners per accent and context. (PDF) [file pone.0231089.s008.pdf]

S8 Table. Germany speech understandability and speaker evaluations per accent (British English, American English, Dutch English; 1=negative; 3=neutral; 5=positive) and context (Lecture, Audio Tour, Job Pitch)

	**Speech understandability**			**Speaker evaluations**		
**Accent, Context**	Intelligibility Mean (SD)	Comprehensibility % correct	Interpretability % correct	Status Mean (SD)	Affect Mean (SD)	Dynamism Mean (SD)
**Dutch English Lecture[a]**	7.22(3.61) n=114	91.2% n=104	76.3% n=87	3.83(.70) n=114	3.55(94) n=114	3.39(1.12) n=114
**Dutch English Audio Tour[a]**	7.68(3.64) n=59	72.9% n=43	78% n=46	3.75(.60) n=59	3.27(.69) n=59	3.29(.99) n=59
**Dutch English Job Pitch[b]**	7.37(3.80) n=52	80.8% n=42	75% n=39	3.34(.94) n=52	3.13(.93) n=52	2.77(.98) n=52
**British English Lecture**	7.67(3.93) n=49	93.9% n=46	89.8% n=44	3.91(.78) n=49	3.35(.93) n=49	3.37(.92) n=49
**British English Audio Tour**	7.38(3.91) n=48	77.1% n=37	79.2% n=38	3.71(.72) n=48	3.09(.89) n=48	3.04(1.04) n=48
**British English Job Pitch**	6.31(4.11) n=87	78.2% n=68	70.1% n=61	3.72(.73) n=87	3.22(1.07) n=87	3.16(1.06) n=87
**American English Lecture**	8.34(.25) n=53	84.9% n=45	86.8% n=46	3.81(.67) n=53	3.18(.94) n=53	3.26(1.04) n=53
**American English Audio Tour**	8.01(4.01) n=102	81.4% n=83	76.5% n=78	3.84(.71) n=102	3.56(.87) n=102	3.28(1.00) n=102
**American English Job Pitch**	7.83(3.67) n=53	84.9% n=45	83% n=44	3.66(.56) n=53	2.89(.93) n=53	3.23(.94) n=53

[a]max. 11 words; [b]max. 12 words intelligible. N=617; n= number of listeners per accent and context.
