# Supplementary material for: Where does a ‘foreign’ accent matter? German, Spanish and Singaporean listeners’ reactions to Dutch-accented English, and standard British and American English accents
Source: PLoS One. 2020 Apr 29;15(4):e0231089. doi: 10.1371/journal.pone.0231089 (PMC7190091; doi:10.1371/journal.pone.0231089)
Supplement: S4 Table — ᵃmax. 11 words; ᵇmax. 12 words intelligible. N = 542; n = number of listeners per accent and context. (PDF) [file pone.0231089.s009.pdf]

S9 Table. Singapore speech understandability and speaker evaluations per accent (British English, American English, Dutch English; 1=negative; 3=neutral; 5=positive) and context (Lecture, Audio Tour, Job Pitch)

	**Speech understandability**			**Speaker evaluations**		
**Accent, Context**	Intelligibility Mean (SD)	Comprehensibility % correct	Interpretability % correct	Status Mean (SD)	Affect Mean (SD)	Dynamism Mean (SD)
**Dutch English Lecture[a]**	6.12(3.84) n=59	83.1% n=49	86.4% n=51	3.71(.60) n=59	3.45(.72) n=59	3.12(.82) n=59
**Dutch English Audio Tour[a]**	6.29(3.55) n=63	85.7% n=54	69.8% n=44	3.87(.50) n=59	3.67(.65) n=59	3.49(.95) n=59
**Dutch English Job Pitch[b]**	6.66(4.12) n=58	65.5% n=38	69% n=40	3.78(.64) n=58	3.52(.96) n=58	3.41(1.06) n=58
**British English Lecture**	7.20(4.10) n=64	89.1% n=57	82.3% n=53	3.98(.63) n=64	3.41(.66) n=64	3.06(.97) n=64
**British English Audio Tour**	7.07(3.77) n=60	78.3% n=47	83.3% n=50	3.96(.66) n=60	3.54(.81) n=60	3.40(.92) n=60
**British English Job Pitch**	7.68(3.97) n=63	68.3% n=43	71.4% n=45	3.55(.68) n=63	2.96(.91) n=63	3.03(.95) n=63
**American English Lecture**	7.19(4.31) n=62	85.5% n=53	83.9% n=52	3.94(.57) n=62	3.36(.64) n=62	3.13(.88) n=62
**American English Audio Tour**	6.60(4.40) n=58	81% n=47	74.1% n=43	3.52(.69) n=58	3.16(.84) n=58	3.00(.88) n=58
**American English Job Pitch**	6.69(4.66) n=55	74.5% N=41	72.7% n=40	3.73(.51) n=55	3.26(.85) n=55	3.11(.92) n=55

[a]max. 11 words; [b]max. 12 words intelligible. N=542; n= number of listeners per accent and context.
