# Supplementary material for: Where does a ‘foreign’ accent matter? German, Spanish and Singaporean listeners’ reactions to Dutch-accented English, and standard British and American English accents
Source: PLoS One. 2020 Apr 29;15(4):e0231089. doi: 10.1371/journal.pone.0231089 (PMC7190091; doi:10.1371/journal.pone.0231089)
Supplement: S5 Table — ᵃmax. 11 words; ᵇ max. 12 words intelligible. N = 540; n = number of listeners per accent and context. (PDF) [file pone.0231089.s010.pdf]

S10 Table. Spain speech understandability and speaker evaluations per accent (British English, American English, Dutch English; 1=negative; 3=neutral; 5=positive) and context (Lecture, Audio Tour, Job Pitch)

	**Speech understandability**			**Speaker evaluations**		
**Accent, Context**	Intelligibility Mean (SD)	Comprehensibility % correct	Interpretability % correct	Status Mean (SD)	Affect Mean (SD)	Dynamism Mean (SD)
**Dutch English Lecture[a]**	5.23(3.10) n=61	98.4% n=60	90.2% n=55	4.06(.54) n=61	3.65(.74) n=61	3.39(.95) n=61
**Dutch English Audio Tour[a]**	7.26(3.16) n=61	83.6% n=51	83.6% n=51	4.03(.59) n=61	3.73(.69) n=61	3.54(.79) n=61
**Dutch English Job Pitch[b]**	5.33(2.91) n=60	76.7% n=46	65% n=39	3.72(.74) n=60	3.35(.84) n=60	3.12(1.03) n=60
**British English Lecture**	6.12(2.78) n=60	88.3% n=53	90% n=54	4.13(.759) n=60	3.73(.67) n=60	3.38(.82) n=60
**British English Audio Tour**	7.07(2.73) n=60	76.7% n=46	78.3% n=47	4.05(.60) n=60	3.48(.70) n=60	3.42(1.01) n=60
**British English Job Pitch**	5.37(3.32) n=60	70% n=42	61.7% n=37	3.59(.58) n=60	3.05(.90) n=60	3.12(1.06) n=60
**American English Lecture**	7.09(3.47) n=59	91.5% n=54	93.2% n=55	3.69(.65) n=60	3.29(.75) n=60	3.13(.96) n=60
**American English Audio Tour**	6.98(3.10) n=60	93.3% n=56	83.3% n=50	3.77(.72) n=59	3.31(.67) n=59	3.24(.90) n=59
**American English Job Pitch**	6.37(3.16) n=60	78% n=46	59.3% n=35	3.74(.68) n=59	3.08(.83) n=59	2.88(1.04) n=59

[a]max. 11 words; [b] max. 12 words intelligible. N=540; n= number of listeners per accent and context.
